# The giant panda gut harbors a high diversity of lactic acid bacteria revealed by a novel culturomics pipeline

**DOI:** 10.1128/msystems.00520-24

**Published:** 2024-06-26

**Authors:** Wenping Zhang, Lijun Zheng, Junjin Xie, Xiaoyan Su, Mingchun Zhang, He Huang, Stephan Schmitz-Esser, Shizhang Du, Yu Yang, Jiqin Xie, Qinrong Zhang, Shuran Yu, Qiang Guo, Hairui Wang, Liang Zhang, Kong Yang, Rong Hou

**Affiliations:** 1 Key Laboratory of Monitoring Biological Diversity in Minshan Mountain of National Park of Giant Pandas at Mianyang Teachers' College of Sichuan Province, College of Life Science and Biotechnology, Mianyang Normal University, Mianyang, Sichuan, China; 2 Sichuan Key Laboratory of Conservation Biology on Endangered Wildlife, Chengdu Research Base of Giant Panda Breeding, Chengdu, Sichuan, China; 3 China Conservation and Research Center for the Giant Panda, Chengdu, Sichuan, China; 4 Department of Animal Science, Iowa State University, Ames, Iowa, USA; 5 Institute of Qinghai-Tibetan Plateau, Southwest Minzu University, Chengdu, Sichuan, China; The University of Maine, Orono, Maine, USA

**Keywords:** giant panda, lactic acid bacteria, culturomics, SCFAs

## Abstract

Some lactic acid bacteria (LAB) can provide significant health benefits, which are critically important for the conservation of endangered animals, such as giant pandas. However, little is known about the diversity and culturability of LAB in the giant panda gut microbiota. To understand the roles of LAB in giant panda conservation, it is critical to culture bacterial strains of interest. In this study, we established a pipeline to culture bacterial strains using enrichment of target bacteria with different liquid media and growth conditions. Then, the strains were isolated in solid media to study their functions. Using 210 samples from the culture enrichment method and 138 culture-independent samples, we obtained 1120 amplicon sequencing variants (ASVs) belonging to *Lactobacillales*. Out of the 1120 ASVs, 812 ASVs from the culture enrichment approach were twofold more diverse than 336 ASVs from the culture-independent approach. Many ASVs of interest were not detected in the culture-independent approach. Using this pipeline, we isolated many relevant bacterial strains and established a giant panda gut bacteria strain collection that included strains with low-abundance in culture-independent samples and included most of the giant panda LAB described by other researchers. The strain collection consisted of 60 strains representing 35 species of 12 genera. Thus, our pipeline is powerful and provides guidance in culturing gut microbiota of interest in hosts such as the giant panda.

**IMPORTANCE:**

Cultivation is necessary to screen strains to experimentally investigate microbial traits, and to confirm the activities of novel genes through functional characterization studies. In the long-term, such work can aid in the identification of potential health benefits conferred by bacteria and this could aid in the identification of bacterial candidate strains that can be applied as probiotics. In this study, we developed a pipeline with low-cost and user-friendly culture enrichment to reveal the diversity of LAB in giant pandas. We compared the difference between culture-independent and culture enrichment methods, screened strains of interest that produced high concentrations of short-chain fatty acids (SCFAs), and we investigated the catalog of virulence factors, antibiotic resistance, butyrate and lactate synthesis genes of the strains at a genomic level. This study will provide guidance for microbiota cultivation and a foundation for future research aiming to understand the functions of specific strains.

## INTRODUCTION

The giant panda (*Ailuropoda melanoleuca*) is a rare and large endemic species in China and is one of the most adored and protected species worldwide. At present, there are more than 600 captive giant pandas according to the studbook of giant pandas of 2020 reported by the Chinese Association of Zoological Gardens. More than 1,800 wild giant pandas are found in the mountainous areas of Sichuan, Shaanxi, and Gansu provinces of China according to the Fourth National Survey of Giant Pandas completed in 2015. The threat level of giant pandas has been downgraded from "endangered" to "vulnerable" (http://www.forestry.gov.cn). However, giant pandas are often threatened by intestinal diseases, the main reason for mortality caused by ascarid nematodes and bacterial infections, such as *Escherichia coli*, *Klebsiella pneumoniae*, *Clostridium perfringens* (1–4). Thus, it is critical to identify efficient ways to prevent and cure intestinal diseases of this beloved species.

Some lactic acid bacteria (LAB) play a key role for the host animal by providing health benefits. Animal studies have shown that LAB significantly prolonged the life span of *Caenorhabditis elegans* (5) and enabled blood urate control in mice (6). LAB could potentially help to control some cancers (7, 8) and regulate the normal gastrointestinal microbiota, maintain the microecological balance, inhibit pathogen proliferation, and prevent inflammation (9–11).

Owing to the important physiological role of intestinal LAB, screening functional LAB from giant pandas is a good way to potentially use them as probiotics and to prevent and treat intestinal diseases. As of now, the isolated LAB strains from giant pandas mainly included *Bifidobacterium* (12), *Lactobacillus* (11, 13–16), and *Weissella* (17, 18). In these studies, some authors reported the characteristics and drug sensitivity of these strains (12, 17, 18), their survival at low pH and high bile salt concentrations, their activity against pathogens, and their ability to alleviate the inflammatory response (11, 13–15). However, more knowledge about LAB in giant pandas is needed to aid in conservation of giant pandas. A good way to analyze the diversity of LAB and to screen functional strains in giant pandas is to culture the microbiota with culturomics (19).

Cultivation is necessary to screen strains to experimentally investigate microbial traits, to confirm the activities of novel genes through functional characterization studies, and to identify potential health benefits for the development of probiotics (19–21). Culturomics is a comprehensive culture-based approach that applies multiple cultivation conditions to identify bacteria and genes that contribute to potential functions of interest (22, 23). The culturomics was used to successfully culture lower-abundance bacteria (24) and “uncultivable” bacteria (25) in human gut, and to unveil members of the swine gut microbiota (26).

In addition, symbiotic gut bacteria produce different classes of metabolites, such as short-chain fatty acids (SCFAs), which can not only serve as energy substrates but also as signaling molecules with diverse functional roles, including protection of the gut barrier function, modulation of energy metabolism, alteration of immune mechanisms, regulation of metabolic homeostasis, and neuroprotective effects for combating disease and improving health (27, 28).

Thus, in this study, we used culture-independent and culture-enriched methods to reveal the diversity of LAB in giant pandas, compare the difference between the two methods, and establish a pipeline to screen novel strains with the potential for use as probiotics following the combination of liquid and solid cultures. This study provides guidance for future giant panda gut microbiota cultivation.

## RESULTS

### The diversity of the fecal giant panda microbiome based on culture-independent methods

**Fig 1:**
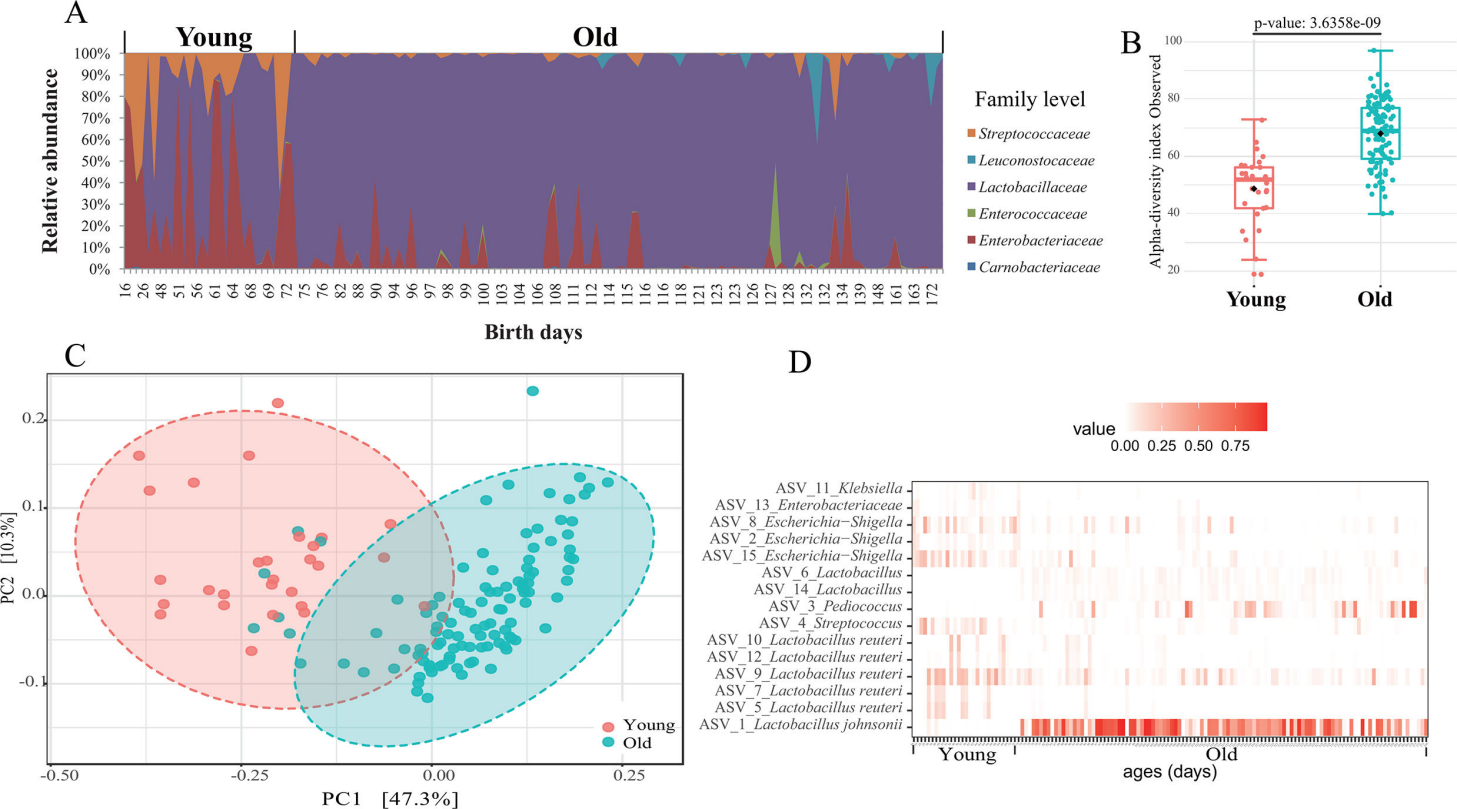
Giant panda gut microbial communities based on culture-independent analyses. (**A**) Relative abundance of gut microbial communities at family level from groups Young to Old, different colors represent different taxonomic families. (**B**) Comparisons of alpha-diversity between groups Young and Old by Wilcoxon signed-rank test. The lines and squares inside boxes represent the median and mean number of observed ASVs, respectively. (**C**) Principal coordinate analysis (PCoA) using unweighted UniFrac distances of 16S rRNA gene sequencing data. The percentage of variation explained by the plotted principal coordinates is indicated on the axes. (**D**) Heatmap of the 15 ASV-level phylotypes identified as key variables for differentiation between Young and Old groups of the giant panda gut microbiota. The values are based on the relative abundance of ASVs. Each column represents one sample.

To characterize the taxonomic profile of LAB from the gut of giant pandas, we followed the pipeline shown in Fig. S1 and sequenced the V3-4 region of the 16S rRNA gene of 138 fecal samples using LAB-specific primers (29). After quality filtering, 5,158,958 high-quality 16S rRNA gene sequences, with an average of 37,384 per sample, were retrieved and ranged from 7,081 to 80,265 for the 138 fecal samples from 13 young giant pandas with an age ranging from 16 to 210 days (Table S1). These 5,158,958 reads were grouped into 880 ASVs and were assigned to two phyla (*Firmicutes* and *Proteobacteria*) and six families. On average, out of the six families, more than 96% of the bacterial sequences derived from *Lactobacillaceae* and *Enterobacteriaceae* (Fig. 1A). Two distinct community configurations were demarcated by these data and marked shifts in abundant lineages around the second month of life (about 72 days old) which seemed to follow dietary adjustments (Fig. 1A). The culture-independent fecal samples from giant pandas less than 72 days old were denoted as group “Young” and the remaining samples were denoted as group “Old”. From birth to seven months of age, the relative abundances of *Lactobacillaceae* increased on average (Young: 48.9% ± 18.0%; Old: 91.7% ± 2.0%; *P* < 0.000001) and that of *Enterobacteriaceae* decreased on average (Young: 33.8% ± 10.7%; Old: 5.3% ± 1.2%; *P* < 0.00001, paired *t*-tests; Fig. 1A). These results were consistent with Zhang et al. (30).

At the genus level, six taxa were each found at >1% relative abundance and their overall abundances were as follows: *Lactobacillus* (76.7%) *> Pediococcus* (7.9%) *> Escherichia-Shigella* (5.6%) > *Streptococcus* (3.5%) *> Klebsiella* (2.6%) *> Lactococcus* (1.2%). Among these genera, *Lactobacillus* was the only genus with a positive relationship with age from birth to seven months of age, *Escherichia-Shigella* and *Streptococcus* had a negative relationship with age from Young to Old (Fig. S2). In addition, about 1.5% of the total sequences could not be assigned to any genus (Fig. S2).

The number of observed ASVs increased with giant panda age and the Old group had a significantly higher α-diversity than the Young group (*t*-test, *P* < 0.0001; Fig. 1B). However, the Shannon diversity indices were similar in the two groups, which showed wider abundance variations of ASVs in the Old group compared to the Young group (Table S1).

The principal coordinate analysis (PCoA) of unweighted UniFrac distances (sensitive to rarer taxa) showed two clear clusters and 47.3% variability contributed to the difference between the Young and Old groups. The PCoA of weighted UniFrac distances (sensitive to abundances of taxa) showed only 25.4% variability contributed to the difference between Young and Old, with a larger overlap than that of unweighted UniFrac distances between Young and Old (Fig. S3). These results were consistent with Zhang et al. (30) and Xue et al. (31). Machine learning techniques (random forest) showed that 15 ASVs could be used to reliably discriminate samples of the Young and Old groups (Fig. 1D). Four of these discriminatory ASVs were overrepresented in Old and included 3 ASVs belonging to *Lactobacillus*. The remaining 11 ASVs were overrepresented in the Young samples and five out of the 11 ASVs belonged to *Lactobacillus reuteri* (Fig. 1D). These results showed that giant pandas of different ages were characterized by different LAB.

### The diversity of the giant panda fecal microbiota based on a culture-enriched approach

A total of 210 samples from culture-enriched samples (Fig. S1) were used to analyze bacterial diversity with liquid culture media. The 210 samples were cultured from fecal samples of cubs (<1 year old, denoted by YZ), subadult (1–3 years old, denoted by WC) and adult (>4 years old, denoted by CN) giant pandas using 33 media (Table S2). To assess the results of the cultivation-based approach, the cultured samples were characterized by 16S rRNA gene amplicon sequencing using general primers of V3-4 region. A total of 21,864,360 high-quality reads were generated, with an average of 64,423 per sample, and were grouped into 5,130 ASVs. The relative abundance of two phyla (*Firmicutes*, 83.5%; *Proteobacteria*, 13.9%) and four families (*Lactobacillaceae*, 69.7%; *Enterobacteriaceae*, 12.4%; *Streptococcaceae*, 8.5%; and *Enterococcaceae*, 1.9%) were highest.

**Fig 2:**
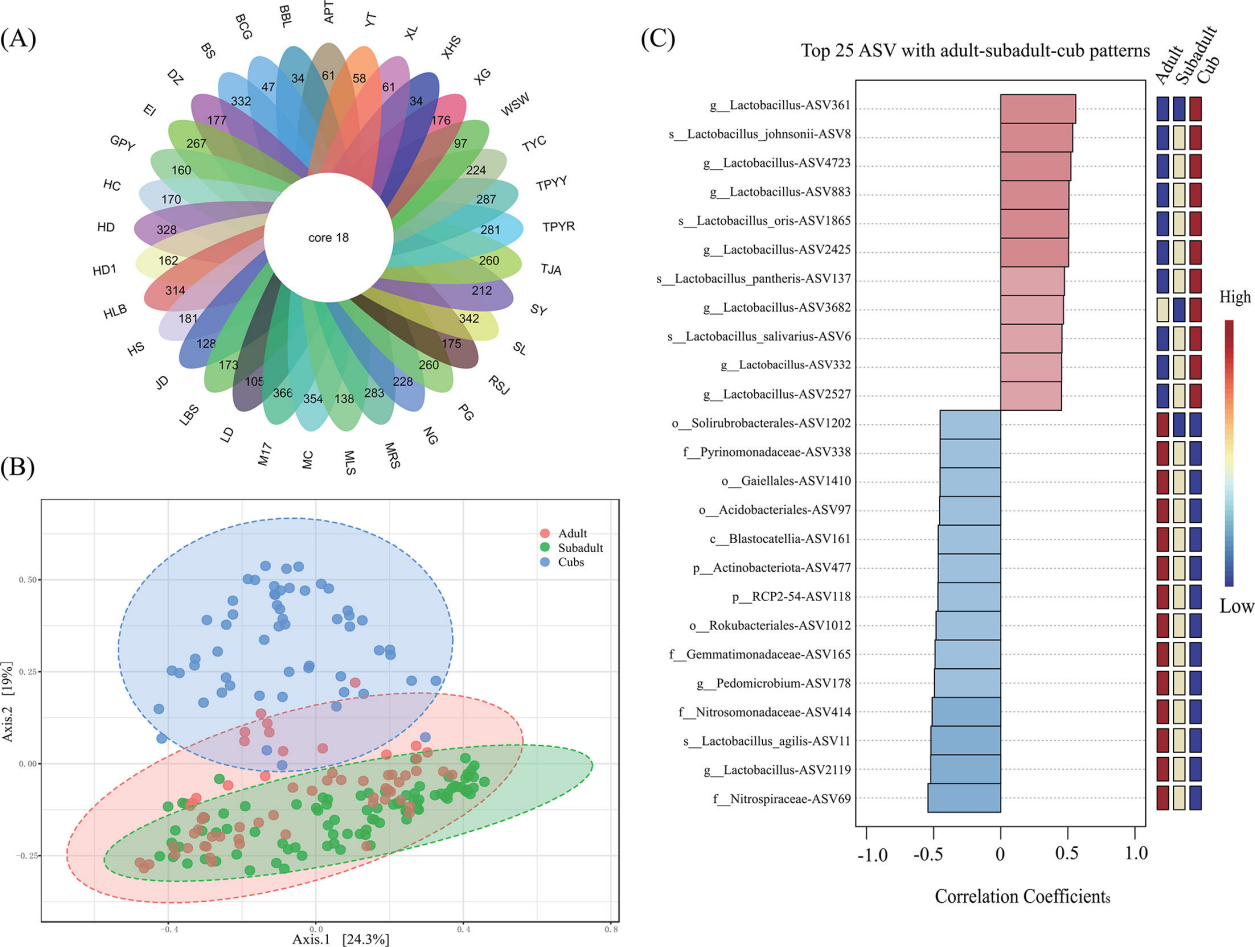
Giant panda gut microbial communities based on culture enrichment approaches. (**A**) Venn diagram at genus level for all culture media. Here, the number shown at every medium indicated the count of all genera retrieved in the medium. The full names of the media can be found in Table S2. (**B**) PCoA using Bray-Curtis distances of 16S rRNA data showing the culture-enriched microbiota separated by ages in the second axis (PERMANOVA; F-value: 35.379; *R*^2^: 0.25475; *P* value < 0.001). The percentage of variation explained by the plotted principal coordinates is indicated on the axes. (**C**) Correlation coefficients of the top 25 ASVs relative abundance among the groups adult, subadult, and cubs. Red represents high and blue low abundance.

At genus level, the 33 culture media cultured 695 genera with *Lactobacillus* being the most abundant. Among those genera, 18 genera, including *Lactobacillus*, *Enterococcus*, *Streptococcus*, *Escherichia-Shigella*, *Lactococcus*, *Pediococcus*, *Weissella*, *Yersinia*, *Leuconostoc*, *Clostridium*_*sensu*_*stricto*_1, *Bifidobacterium*, *Citrobacter*, *Morganella*, *Providencia*, *Turicibacter*, *Klebsiella*, *Raoultella*, and *Terrisporobacter*, were grew in all culture media (Fig. 2A). Within the 33 culture media, six culture media (BS, SL, M17, HLB, HD, and MC, more details in Table S3) cultured more than 300 genera, yet six culture media (BCG, BBC, APT, CL, XHS, and WSW) cultured less than 100 genera (Fig. 2A). Notably, more than 1700 ASVs were cultured from M17 representing the highest diversity among all culture media (Fig. S4; Table S3). Forty-nine ASVs were common among the 33 culture media (Fig. S4).

PCoA of Bray-Curtis distances indicated a clear clustering by age, which showed 19% variability contributed to the difference between cubs and adult or subadult (PERMANOVA; F-value: 35.379; *R*^2^: 0.255; *P* value < 0.001) and no difference existed between subadult and adult (Fig. 2B), which is consistent with the PCoA of unweighted UniFrac distances (*R*^2^ = 0.187), but the correlation was weak among age groups of weighted UniFrac distances (*R*^2^ = 0.054) (Fig. S5). Thus, although the media in this study were designed to enrich LAB, the difference between cubs and other groups mainly derived from the observed bacterial ASVs, which is consistent with the culture-independent approach (Fig. 1) and the results of Zhang et al. (30). The correlation coefficients of adult-subadult-cub patterns showed that cubs were enriched in *Lactobacillus* (Fig. 2C). The effect of the duration of cultivation on culture enrichment-associated microbiota was relatively small (*R*^2^ <0.1) compared to the effect of age (Fig. S6).

### Correlation between SCFAs and bacteria

**Fig 3:**
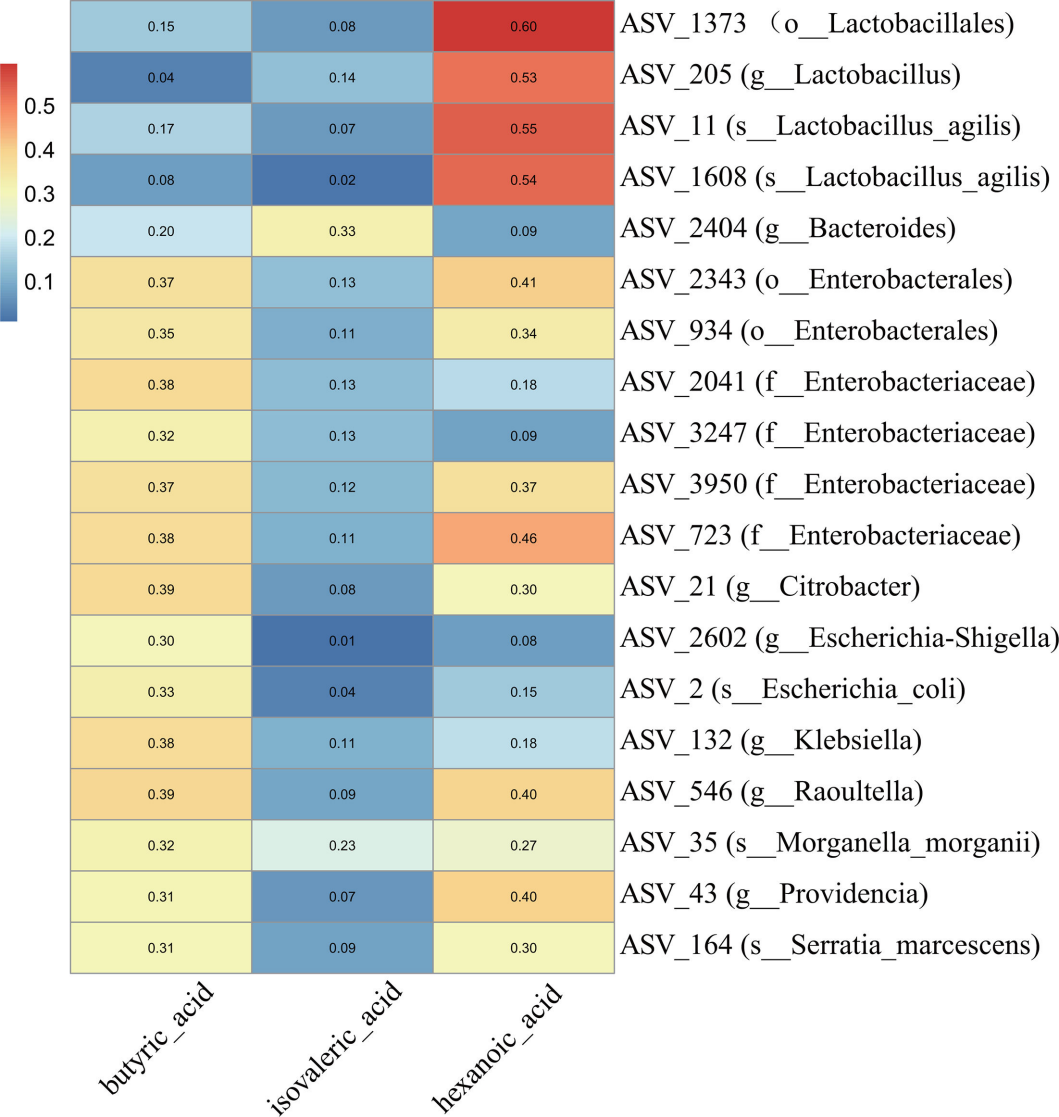
Heatmap showing Spearman’s correlations of different ASVs in culture enrichment with SCFA concentration. The number indicates the correlations (*R*^2^). Only the positive correlations between the important ASVs and SCFAs (*P* < 0.05) are shown.

We next sought to identify individual microbial ASVs that could be used to discriminate the concentrations of SCFAs. After excluding the low abundance ASVs (mean < 0.001% and the max <0.1%), 326 ASVs of cultured samples were used for the correlation analysis. The Shapiro-Wilk normality test showed that the relative abundance of ASVs and the concentration of SCFAs in this study were significantly deviated from normal distribution (*P* < 0.0001) and Spearman rank correlation coefficients were used to assess the correlation between taxonomic relative abundance with increasing concentration of each SCFAs and the level of significance was kept at the default of *P* < 0.05. A total of 19 ASVs showed significant positive correlation with either butyric acid (14 ASVs, 0.3 < *R*^2^ <0.4), isovaleric acid (1 ASV, *R*^2^ = 0.33), or hexanoic acid (4 ASVs, 0.5 < *R*^2^ <0.6; 8 ASVs, 0.3 < *R*^2^ <0.5) (*P* < 0.00001) (Fig. 3). Out of these ASVs, all four ASVs with medium correlations with hexanoic acid belonged to *Lactobacillales*, specifically to *Lactobacillus agilis*; and the 14 ASVs with weak correlation with butyric acid or hexanoic acid belonged to *Enterobacteriales* (Fig. 3).

### Bacterial interaction networks

**Fig 4:**
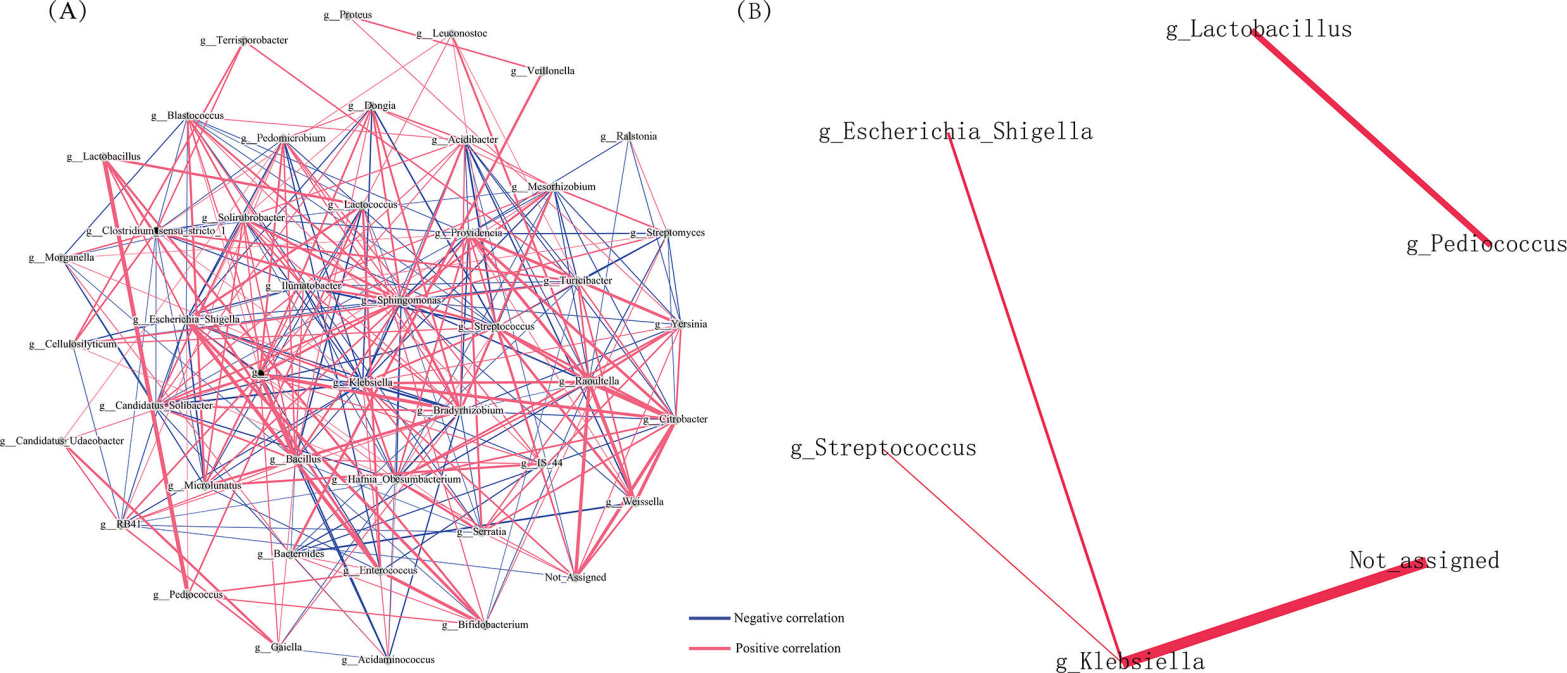
Genus-level bacterial correlation networks of culture enrichment (**A**) and culture-independent approaches (**B**). Nodes represent genera. Blue and red edges represent averaged positive and negative inter-species interaction coefficients across all species associated with the given genus. Line width represents the magnitude of the averaged interaction and only the high confidence interactions (*P* < 0.01) with high absolute correlation coefficients (>0.3) were presented.

To infer potential interactions among the microbiota communities in this study, co-occurrence networks of bacterial species were constructed based on taxonomic distribution (only including major species with over 0.1% average relative abundance). Strong positive correlations were found between *Lactobacillus* and *Pediococcus*, between *Escherichia_Shigella* and *Klebsiella*; between *Klebsiella* and *Streptococcus* both in the culture-independent and culture enrichment approaches. In culture enrichment, the dominant genus *Lactobacillus* also showed strong negative correlation with *Bifidobacterium* (*r* = 0.52; *P* = 0.0099), *Enterococcus* (*r* = 0.56; *P* = 0.0099), *Escherichia_Shigella* (*r* = 0.43; *P* = 0.0099), and *Lactococcus* (*r* = 0.57; *P* = 0.0099) (Fig. 4). *Sphingomonas* represented the maximum node in the network, which had negative correlation with *Streptococcus* and *Klebsiella*, revealing potentially inhibitory actions, and had positive correlations with other bacteria, such as *Bradyrhizobium* and *Solirubrobacter*, revealing potential mutual enhancement of bacteria (Fig. 4).

### Comparison of LAB between culture-independent and culture-enriched methods

**Fig 5:**
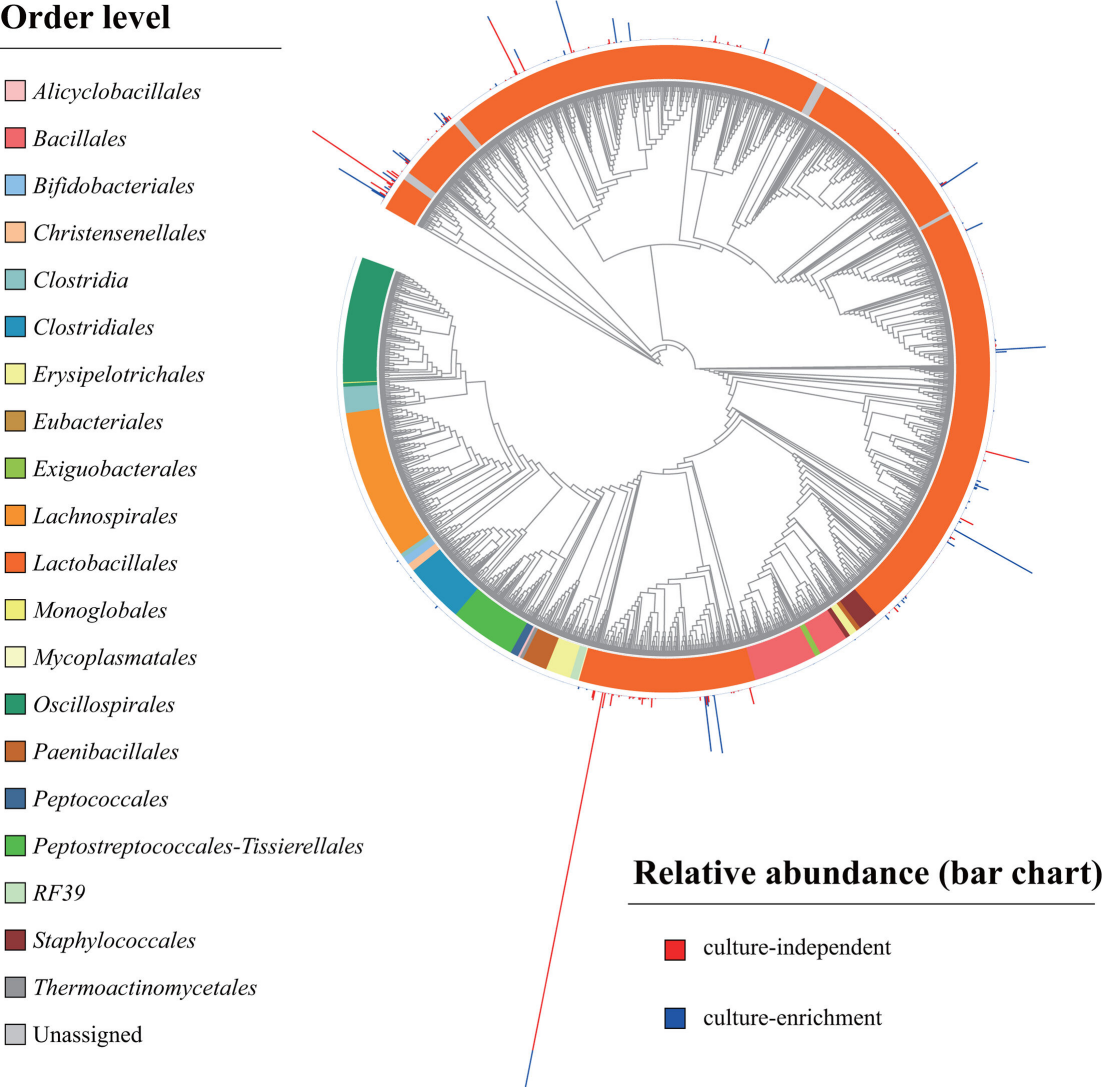
Taxonomic dendrogram of *Actinobacteria*, *Bacilli* and *Clostridia* from culture-independent and culture-enriched methods. Color ranges identify order within the tree. Colored bars represent the relative abundance of each ASV in the two methods. The taxonomic dendrogram was generated with one representative sequence of each ASV using Unipro UGENE and displayed with the use of iTOL. Total relative abundances, colors, and taxa of all ASVs are listed in Table S4.

The overlapping region (336 bp) of reads between culture-independent and culture-enriched (group cubs) samples was used to compare their microorganisms from the classes *Actinobacteria*, *Bacilli* and *Clostridia* using qiime2 (32). A total of 1713 ASVs was found from the three classes. No *Actinobacteria* and *Clostridia* ASVs were found in the culture-independent approach due to the LAB-specific primers, and 1310 ASVs belonged to *Bacilli* that included 11 orders whose number of ASVs ranged from one (*Thermoactinomycetales*, *Mycoplasmatales*) to 1120 (*Lactobacillales*) (Fig. 5; Table S4). Within these 11 orders, the culture-independent approach detected ASVs only in the *Lactobacillales* (Fig. 5; Table S4). Out of the 1120 *Lactobacillales* ASVs, 812 ASVs from the culture enrichment were twofold more diverse than 336 ASVs from the culture-independent approach and only 28 ASVs were shared between the two methods, which showed that approximately 73% of the total ASVs were culturable (Table S4; Fig. 5).

A total of 13 genera belonging to the *Lactobacillales* order, comprised of 1120 ASVs, were observed (Fig. 5; Table S4). Approximately 92% (12/13) of total genera were culturable. Seven genera, including *Enterococcus*, *Lactobacillus*, *Lactococcus*, *Leuconostoc*, *Pediococcus*, *Streptococcus*, and *Weissella*, were found both in the culture enrichment and culture-independent approaches. Among the seven genera, *Lactobacillus* had the highest diversity with 219 ASVs in the culture independent approach and 583 ASVs in the culture enrichment. No shared ASVs between the culture enrichment and culture-independent approach were observed for *Enterococcus*, *Lactococcus*, and *Streptococcus*. The shared ASVs between the two methods included 23 ASVs in the genus *Lactobacillus*, one in *Leuconostoc*, two in *Pediococcus*, and one in *Weissella*. Out of these shared ASVs, only six ASVs had relative abundances higher than 0.1% (Fig. 5; Table S4). In addition, 72 ASVs were unassigned or uncultured at genus level, which indicated the presence of unknown bacteria of *Lactobacillales* in giant pandas (Table S4).

At species level, a total of 38 species were found (Fig. 5; Table S4). *Lactobacillus mucosae* contained the most of the observed ASV diversity with 17 ASVs, followed by *L. agilis* with 13 ASVs, *L. songhuajiangensis* with 7 ASVs, *L. faecis* with five ASVs. *L. agilis* was not found in culture-independent and *L. songhuajiangensis* was not found in culture enrichment (Fig. 5; Table S4).

### The genomes of the isolates from culture enrichment

The samples of M17 medium for culture enrichment included the significantly highest concentration of SCFAs, especially for propionic acid, butyric acid, and isovaleric acid, among the 33 media used (Fig. S7); thus, most of the colonies from M17 medium were used for genome sequencing.

In total, we obtained an assembly of 189  Mb in length with N50 and N90 values of 348 kbp and 33 kbp, respectively. The mean read length was 74,191 base pairs from Nanopore sequencing.

**Fig 6:**
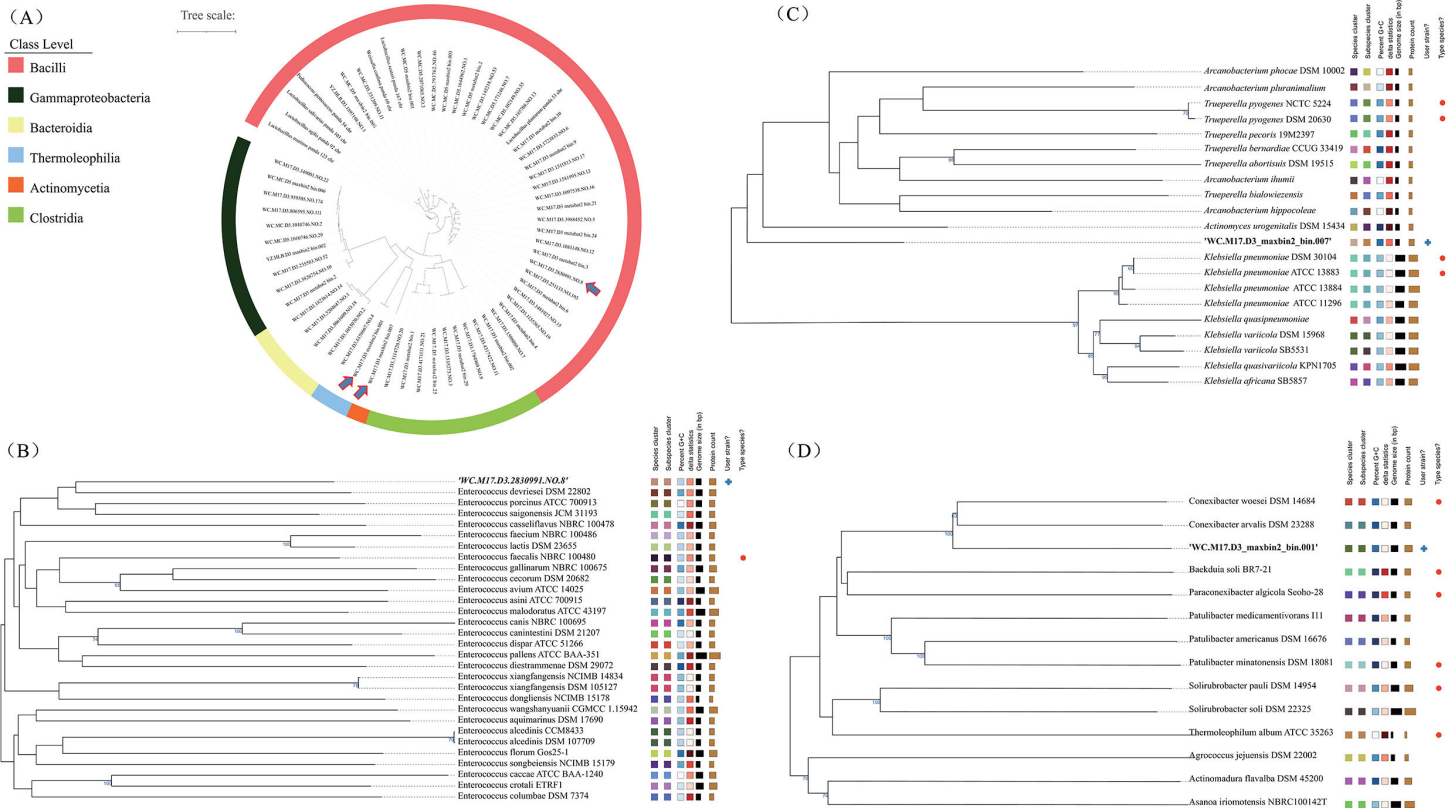
The phylogenetic relationship of select MAGs. Phylogenetic distribution of the MAGs cataloged using GTDB-TK (A) and three putative novel species identified by TYGS and ANI value (<0.95) (B–D) in the culture-enriched approach. The colors in the outer circle of panel A denoted class level of gut microbiome and the arrows in panel A indicated the three putative novel species displayed in more details in panels B, C, and D. The trees shown in panels B, C, and D were downloaded from TYGS (https://tygs.dsmz.de) after uploading the corresponding genome data. The colored boxes to the right of panels B, C, and D, respectively, represent species cluster, subspecies cluster, percent G+C content, delta statistics, genome size (in bp), protein count, what a user strain is, and type species.

The smallest bacterial genomes have a size of approximately 0.1 Mbp (33) and thus, we chose 0.1 Mbp as cutoff to analyze the assembled contigs and bins. In total, we obtained 133 non-redundant contigs and 20 bins larger than 0.1 Mbp. Out of these 153 genomes, 109 were assigned to two phyla (*Firmicutes* and *Proteobacteria*) according to the available reference genomes in NCBI. In addition, we obtained a broad mix of genomes from species from diverse phyla including *Firmicutes*, *Proteobacteria*, *Bacteroidetes*, and *Actinobacteria* according to the Genome Taxonomy Database (GTDB) from the 153 genomes. Out of the 153 genomes, 18 were successfully classified to the genus level and 46 were classified to the family level using GTDB. For each of these 64 genomes that were classified by GTDB, we listed the name (colored according to corresponding class) and illustrated its phylogenetic relationships with other species with a dendrogram in Fig. 6A. Several well-known families of the giant panda gut microbiome (Fig. 1 and 5) were abundant in our sequenced genomes, including *Lactobacillaceae* and *Enterococcaceae* (Fig. 6A).

Following the annotation of NCBI and GTDB, we excluded the genomes of *Enterobacteriaceae* that did not belong to LAB and 60 genomes remained for further analysis. Among these 60 genomes, the results of JSpecies showed 54 genomes with ANI >0.95 (Table S5) and six genomes with ANI <0.95 (Table S6). Three out of the six genomes with ANI <0.95 were identified as new species by TYGS (Table S6; Fig. 6B through D). WC.M17.D3.2830991.NO.8 was a single contig of 2.8  Mb, which most closely resembled *Enterococcus devriesei* DSM 22802 (Fig. 6B), was predicted to be 95.12% complete with a contamination score of 1.25% by CheckM (Table S6). WC.M17.D3_maxbin2_bin.007 included 29 contigs with total length of 2.65  Mb, had a close relationship with species in the genera *Actinomyces*, *Trueperella*, and *Arcanobacterium* (Fig. 6C), was predicted to be 96.34% complete with a contamination score of 2.93% and the highest ANI was shared with *Trueperella* (Table S6). WC.M17.D3_maxbin2_bin.001 included two contigs with total length of 6.2  Mb, which most closely resembled *Conexibacter* (Fig. 6D), was predicted to be 92.81% complete with a contamination score of 0.93% (Table S6). Together, these results showed that WC.M17.D3_maxbin2_bin.007 represented a new *Trueperella* species and was named as *Trueperella pandaia*; WC.M17.D3.2830991.NO.8 represented a new *Enterococcus* species and was named as *Enterococcus pandaia*; WC.M17.D3_maxbin2_bin.001 represented a new *Conexibacter* species and was named as *Conexibacter pandaia*.

Among the 60 genomes, the genomes of *L. plantarum* had the highest genetic diversity (Table S6). A total of 12 *L*. *plantarum* genomes were found in this study and could be divided into five subspecies clusters according to the results of TYGS (Fig. S8; Table S6). The number of genomes within the five subspecies clusters ranged from 1 to 7 (Fig. S8). Importantly, the pairwise ANI comparisons among these *L. plantarum* genomes ranged between 97% and 100% suggesting the *L. plantarum* genomes represented strain-based variations between each other.

**Fig 7:**
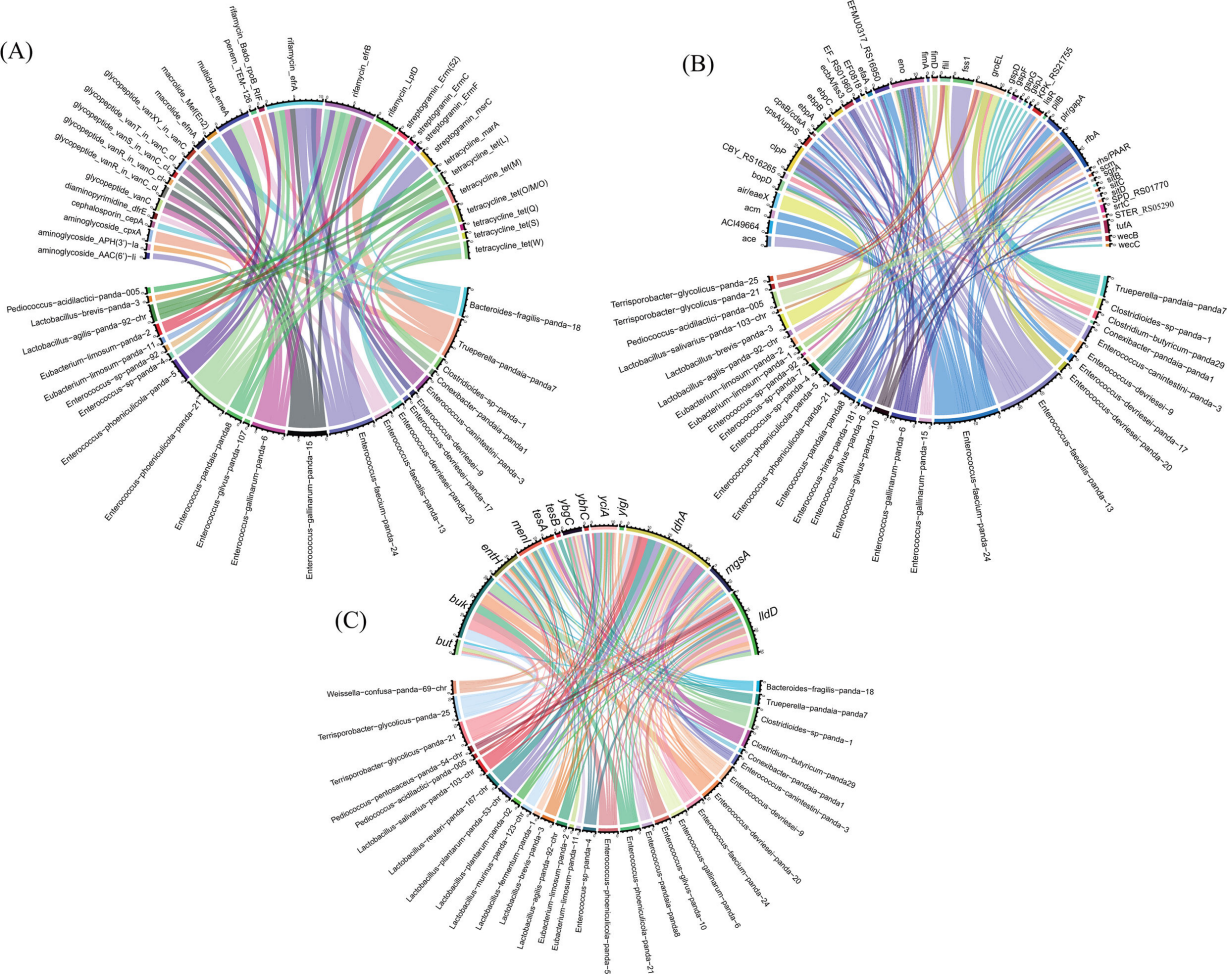
Genomic compositions in the genomes. The distribution of ARG types (**A**), VFDB types (**B**), potential genes contributing to the biosynthesis of butyrate and lactate (**C**), and their counts in the assembly genomes were shown here.

A total of 31 antibiotic resistance genes (ARGs) were found in 23 genomes out of the 60 genomes (Fig. 7A). The rifamycin resistance gene (including *Bado_rpoB_RIF*, *efrA*, *efrB*, *LptD*) had the most gene counts among the annotated ARGs and was present in 12 genomes (Fig. 7A). Notably, more than half ARGs (18 of 31) existed in *Enterococcus* species. In contrast, some *Lactobacillus* species, such as *L. plantarum*, *L. salivarius*, *L. murinus* and *L. reuteri*, did not contain ARGs (Fig. 7A).

By aligning the protein sequences of the 60 genomes against the virulence factor database (VFDB, protein sequences of full data set) (34), 44 virulence genes were determined in 29 out of the 60 genomes (Fig. 7B). Similar to the ARGs, most of the virulence genes (29 of 44) were found in *Enterococcus* species while *Lactobacillus* species, such as *L. plantarum*, *L. salivarius*, *L. murinus* and *L. reuteri*, rarely contained virulence genes (Fig. 7B).

Butyrate kinase (*buk*) and butyryl-CoA:acetate CoA transferase (*but*), which are key enzymes in butyrate formation (35), were used to map high-quality genomes (completeness >80% and contamination <10%) of the 60 genomes. The results showed that *Eubacterium* contained two copies of *but* and other species, such as *Lactobacillus* and *Enterococcus*, did not contain *but*. Louis et al. (36) reported *buk* has high diversity, which is consistent with our results and we found at least 5 copies of *buk* in the genomes of some strains, such as *Clostridioides* sp. panda-1, *Terrisporobacter glycolicus* panda-21 or panda-25, and the strains of *Enterococcus* also contained few copies of *buk* (Fig. S9). Although *buk* and *but* were not found in the gnomes of *Lactobacillus*, one or two copies of *entH*, *ybgC*, *yciA*, and *menI* are contained in the *Lactobacillus* genomes and have a potential contributing to butyrate production (Fig. 7C). Moreover, we found few copies of lactate dehydrogenase (encoded by *ldh*), the major contributor to lactate production (37), in the *Lactobacillus* genomes (Fig. 7C). Similar to *buk*, lactate dehydrogenase A (*ldhA*) has high diversity (Fig. S10). *mgsA* and *lldD* that were related with lactate metabolism also showed in the *Lactobacillus* genomes (Fig. 7C). The *Enterococcus* genomes also contained *ldhA*, *mgsA*, and *lldD* (Fig. 7C).

## DISCUSSION

This study conducted an analysis combining culture-independent and culture-enriched approaches to determine the overall abundance, diversity, and taxonomy of LAB in giant pandas. This approach estimates both cultivable and uncultivable populations of LAB, thus serving as a benchmark estimation of true diversity compared to the findings of the culture-based approach. In this study, we found that the culture enrichment identifies more LAB diversity than the culture-independent approach (Table S4; Fig. 5). This was consistent with other studies, such as a recent study by Ito et al. (25), which recovered 61% of the total ASVs in fecal samples using 26 culturing media. These results also suggest that culturomics is an important complement for metagenomics to gain a thorough insight into the gut microbiota and the cultured-independent and culture-enriched methods should be combined to investigate the diversity of *Lactobacillales* although the culture enrichment has the potential to identify more diversity within the *Lactobacillales*. In addition, several studies have shown that both wild and captive giant pandas undergo a seasonal change in bamboo part preference (culms, shoots, and leaves) (38–40) and also have corresponding shifts in their gut microbiota (31, 41, 42). Thus, future studies that cover more samples from wild giant pandas and from different seasons are necessary to obtain a better insight into the LAB in giant pandas.

We found that some genera of the order *Lactobacillales* were observed only in the culture enrichment, such as *Abiotrophia*, *Atopostipes*, *Carnobacterium*, and *Vagococcus*. Similarly, *L. agilis* was only found in using culture enrichment. One possible reason is that the LAB-specific primers in our cultured-independent approach do not amplify/match those bacteria that were only found in our culture-enriched method. However, the LAB-specific primers should be better suited to detect the LAB diversity than general bacterial primers, as shown in a similar study with the culture-independent approach and the general bacterial primers (43). The study by Liu et al. (43) found a total of 271 ASVs from fecal samples belonged to *Lactobacillales* and out of the 271 ASVs, 145 derived from cubs, 96 from adults, 131 from old, and 109 from young pandas, whose LAB diversity was lower than in our study, which found 336 ASVs in cubs with the culture-independent approach and the LAB-specific primers. Another possible reason might be that specific media could promote the abundance and allow some low-abundance bacteria to reach the detection threshold of PCR and sequencing. Samples of the cultured-independent method contained 10^10^–10^11^ bacteria per gram of sample can be sequenced with deep shotgun sequencing and the less-abundant bacteria can be overlooked (such as bacteria < 10^5^ cells per gram of sample), but samples of culture-enriched method are able to detect 10^2^ bacteria per gram of samples (20, 22, 23).

Out of the ASVs derived from the culture enrichment approach, 19 ASVs had positive correlations with the concentrations of butyric, isovaleric, and/or hexanoic acid based on Spearman rank correlation coefficient (Fig. 3; *R*^2^ <0.3, *P* < 0.05). Among these 19 ASVs, none belonged to genus *Bacteroides*, such as *Bacteroides fragilis*, which has been correlated positively with butyric and propionic acid according to the results of others (44, 45). However, according to the Pearson rank correlation coefficients (Fig. S11), it was revealed that ASV_17 (s__*Bacteroides fragilis*) correlated positively with butyric acid (*R*^2^ >0.5, *P* < 0.05). Kircher et al. (46) found a strong correlation between the average concentration of butyrate and growth of bacteria using the acetyl-CoA pathway. The pathway is present in *Bacteroides fragilis* genome (Bacteroides-fragilis-panda-18, Fig. 7C), which supported the results of the Pearson rank correlation coefficients (Fig. S11). Although a relationship between *Clostridium butyricum* and SCFA was not found in this study, previous studies demonstrated that *Clostridium butyricum* regulates gut homeostasis (47) and improves intestinal barrier function (48). A few studies showed that *B. fragilis* or *C. butyricum* were hardly observed in culture-independent samples of giant pandas with abundances lower than 0.01% (30, 31), while they were prevalent in our culture-enriched samples. *B. fragilis* was high abundance in culture medium M17 with an average relative abundance of 19.2% in cub samples and *C. butyricum* had high abundance in culture medium PG with an average 6.6% relative abundance in adult samples. Thus, our culture-enriched approach was able to detect and characterize bacteria that are less abundant in the microbiota but still play a significant role in maintaining health, such as *B. fragilis* and *C. butyricum*.

The most important result from our study is that we established a pipeline to culture bacterial strains of interest (Fig. S1). First, we cultured bacteria using special bacterial liquid cultivation media to enrich bacterial strains of interest, such as LAB in this study. Then, we identified the functions of the strains, such as whether the strains have the ability to produce high concentration SCFAs in these liquid media, and analyzed the abundance of the strains with 16S rRNA gene. Finally, we isolated the colonies of the strains on solid media using the liquid media as source. The novel step in our pipeline is that liquid media are used to enrich strains of interest, which has a number of advantages, such as the liquid media may allow the isolation of strains that depend on other strains’ metabolites. Liquid media also allow easier analysis of particular functions, such as the ability to produce SCFAs and the abundance by 16S rRNA gene amplicon sequencing, as the high densities of bacteria in the liquid media containing bacteria of interest can be used as an inoculation source for solid media to increase the chance of isolating colonies of the target bacteria. Moreover, our pipeline does not need special devices, is low cost, and is easy to use by most labs with similar research interests. Following the pipeline, we constructed a giant panda gut strain-collection (Tables S5 and S6). According to the genome analysis, the strain-collection consisted of 60 strains representing 35 species of 12 genera and the majority of these strains belonged to the two genera *Lactobacillus* and *Enterococcus*, which covered almost all species of LAB isolated by researchers from giant pandas (10–18) (Tables S5 and S6). Out of the 35 species, only three species, *Weissella confusa*, *Lactobacillus salivarius*, and *Lactobacillus plantarum* were previously reported from giant pandas by other researchers (11, 13–18). *L. plantarum* strains were described in a few studies (11, 13, 14), which was consistent with our results, as we found 12 *L*. *plantarum* strains with high genetic diversity (Fig. S8; Table S6). Thus, the strain-collection will be a valuable resource for the health of giant pandas and we provide a high-throughput, low-cost screening alternative over more tedious metagenomic analyses.

## MATERIALS AND METHODS

### Culture-independent samples for LAB

The giant pandas less than 1-year-old had higher relative abundance and diversity of LAB compared with giant pandas more than 1-year-old (30); thus, a total of 138 fecal samples were collected from October 2015 to January 2016 to investigate the LAB of 13 giant pandas born in 2015 with the culture-independent approach. The ages of these giant pandas ranged from 16 to 210 days during sample collection (Table S1). The diets of these giant pandas were panda breast milk in addition to commercial milk as dietary supplements and no bamboo was found in their feces. The ingestion and health status of each giant panda were monitored daily by veterinarians and giant panda keepers. Fresh fecal samples were collected immediately after defecation, were snap-frozen in liquid nitrogen and stored at −80°C for no more than 6 months before DNA extractions.

### Culture-enriched samples for LAB

Three groups, including cubs (<1 year old, denoted by YZ), subadult (1–3 years old, denoted by WC) and adult (>4 years old, denoted by CN) giant pandas, were used for culture enrichment. Each group included four individuals: two females and two males. The cubs’ samples were collected in October 2015 with milk as diet. The subadult and adult samples were collected in May 2020 with bamboo shoots and leaves as diet. Fresh fecal samples were collected in a sterile anaerobic container immediately after defecation and handled in an anaerobic chamber (Bactron) on ice within half hour after defecation. Each individual fecal sample (around 30 g) per group was mixed and suspended in sterile phosphate buffer saline (PBS) (3–4 times volume of stool pool of the corresponding group) and vortexed for 5 min followed by filtration with three layers gauze to remove larger particles. The filtrate was obtained and the cell pellets were collected by centrifugation at 12,000 × *g* for 5 min. Then, the cell pellets were homogenized and resuspended in sterile 1× PBS with a final concentration of 10^9^ bacteria per milliliter and 1 mL was used to anaerobically inoculate 33 liquid culture media (Table S2). The 33 culture media are based on de Man-Rogosa-Sharpe (MRS) broth supplemented with special carbon and nitrogen sources (Table S2). Autoclaving was applied to most media, only media that can't be treated with high temperature and high pressure were sterilized by filtration (0.22 µm pore size) (Table S2). Anaerobic cultures were incubated in an anaerobic chamber (Bactron-600, SHELLAB, USA) under anaerobic atmosphere (5% H_2_, 5% CO_2_, and 90% N_2_) at 35°C for 7 days to favor the growth of bacteria with longer generation time (49). These liquid culture-enriched samples were chosen for 16S rRNA gene sequencing and metabolic analysis at days 1, 3, 5, and 7 after inoculation (Fig. S1).

### 16S rRNA sequence analysis

Fecal DNA (culture-independent samples) and culture-enriched bacterial DNA were isolated following the process described by Zhang et al. (30). The DNA concentrations of each sample were adjusted to 50 ng/µL for subsequent 16S rRNA gene sequencing.

The LAB-specific primers S-G-Lab-0159-a-S-20 (GGA AAC AG (A/G) TGC TAA TAC CG) and S-G-Lab-0677-a-A-17 (CAC CGC TAC ACA TGG AG) (29) amplified V3-4 region of the 16S rRNA gene of culture-independent samples with a 6 bp barcode unique to each sample for the paired primer. For the culture-enriched analysis, the V3-V4 region of the bacterial 16S rRNA genes was amplified by the general bacterial primers reported by Kozich et al. (50) with a 6 bp barcode. The PCR conditions were 94°C for 4 min, followed by 30 cycles of 94°C for 30 s, 54°C for 30 s and 72°C for 30 s and then 72°C for 5 min. The single amplification was performed in 25 µL reactions with 50 ng template DNA and 1U FastStart Taq DNA Polymerase (Roche). Normalized equimolar concentrations of PCR products were then pooled and sequenced using the Illumina MiSeq PE-250 platform according to the standard protocols from Novogene Biotech Co., Ltd. (Beijing, China).

Microbial raw sequences were merged by FLASH (version 1.2.7) (51) and processed using QIIME2 (version 2021.2) using the DADA2 plugin to denoise and quality filter reads (32), which resulted in high-resolution ASVs and a feature table of ASV counts for subsequent analysis. Taxonomy was assigned to the ASV feature table against the SILVA reference database (version 138) (52) in QIIME2. The BIOM-formatted feature table was uploaded for Microbiome Analyst (https://www.microbiomeanalyst.ca) (53) for Alpha- and Beta-diversity analyses after removing low abundance (minimum count = 4 and prevalence in samples ≤20%) and low variance (10% based on inter-quantile range) features.

### SCFA analysis

SCFA analysis was carried out with the culture-enriched samples. Acetic acid, propionic acid, isobutyric acid, butyric acid, isovaleric acid, valeric acid, hexanoic acid were prepared as reference standard, 2-ethylbutyric acid as internal standard. Sample handling and detection followed a patent invented by Zhang et al. (54) and each sample was analyzed in three parallels with an Agilent Technologies 7890A GC System. Sample preparation involved acidification, centrifugation and filtration of the culture solution followed by direct injection of the supernatant solution onto a DB-FFAP elastic quartz capillary column (30 m × 0.25 mm × 0.25 pm) (Agilent). Nitrogen was used as carrier gas at a constant flow rate of 1.0 mL/min. The initial GC-oven temperature of 50°C was kept for 1 minute and then increased with 15°C/min to 120°C, 5°C/min to 170°C, 15°C/min to 240°C, and 3 min was kept at 240°C. The injector temperature was kept at 250°C.

### Genome sequencing

Some novel bacteria that had a positive relationship with concentration of SCFAs in the liquid culture-enriched method were cultured using the corresponding solid medium to be sequenced using Oxford Nanopore sequencing (Fig. S1). DNA was randomly fragmented by Megaruptor (Diagenode, NJ, USA) and size selected (>10 kb) with Bluepippin and the ends of fragments were repaired, A-linked, ligated with a barcode unique to each individual colony. In order to control sequencing cost, approximately 500 colonies were mixed into one sequencing sample. Finally, the sequencing of the barcoded samples was performed with the PromethION Flow Cell Priming Kit (EXPFLP001.PRO.6, Oxford Nanopore) according to the manufacturer’s instructions by Novogene Co., Ltd. (Beijing, China).

Metagenome assembly was performed with the Flye software (Version: 2.4.2-release, https://github.com/fenderglass/Flye/) (55) with --threads 4, -- meta, -g 5 m after filtering low-quality reads with NanoPlot (Version: NanoPlot 1.18.2) (56) at Q > 7. The contigs were binned with MetaBAT2 (57), CONCOCT (58), and MaxBin2 (59), using read abundance profiles generated with bowtie2 (60) and minimap2 (61) as a proxy for differential coverage. The resulting bins were subjected to metawrap-refine (62) to produce the final bins whose completeness and contamination was assessed using CheckM (v.1.0.5) (63).

For taxonomic assignment of dereplicated contigs or bins, genes were predicted using Prodigal (64), and CAT (65) (settings -sensitive -r 10 and -f 0.3) was used with a DIAMOND (66) database built from proteins in the NCBI non-redundant protein database (version: 2021-01). Non-redundant gene sets were built for all predicted genes using CD-HIT. The clustering parameters were 95% identity and 90% coverage. The longest gene was selected as the representative sequence of each gene set and the taxonomy of the species was obtained as a result of the corresponding taxonomy database of the NR library. In addition, the Genome Taxonomy Database Toolkit (GTDB-Tk) (version 2.1.0) (67) was also used to predict whole genome phylogeny and taxonomic classification of the high-quality dereplicated contigs or bins based on a concatenated data set of 120 universally conserved bacterial single-copy genes. A maximum likelihood phylogenetic tree of the alignment proteins of the 120 genes from GTDB-Tk was constructed with iqtree (68) automatic selection model and 1000 bootstrap replicates, visualized and annotated using iTOL (69).

To identify novel species (Fig. S1), we analyzed these genomes using the program JSpecies (70) online (https://jspecies.ribohost.com/jspeciesws/#analyse) to search the genomes against the GenomesDB reference database to provide the closest reference genomes using the tetra correlation search (TCS). Average nucleotide identity (ANI) values were obtained using pairwise genome comparisons between the genomes obtained in this study and the closest reference genomes. The ANI >0.95 criterion was used to identify matches of the same species. The genomes with ANI <0.95 were reclassified and uploaded to the Type (Strain) Genome Server (TYGS), a bioinformatics platform available at https://tygs.dsmz.de (71, 72). The results were obtained from the TYGS 30 May on 2022.

### Constructing the catalog of virulence factors, antibiotic resistance, butyrate, and lactate synthesis genes

The genomes were also used to determine their potential to be used as probiotics by analyzing virulence factors, antibiotic resistance, butyrate and lactate biosynthesis genes (Fig. S1).

The virulence factor database (VFDB, Protein sequences of full data set; http://www.mgc.ac.cn/VFs/download.htm, downloaded 9 November 2022) (34) was used to identify potential virulence factors. The Comprehensive Antibiotic Resistance Database (CARD) (https://card.mcmaster.ca/) was used to annotate antibiotic resistance genes (ARG) through the software Resistance Gene Identifier (RGI) (73).

To screen for genes involved in butyrate and lactate synthesis, a multi-level approach involving Hidden Markov Models (HMM) was used following the approach of Vital et al. (74). Butyrate kinase (*buk*) and butyryl-CoA:acetate CoA transferase (*but*) serve a major role in butyrate formation (75). In addition, Zhao et al. (37) identified eight genes (*tesB*, *tesA*, *entH*, *ybgC*, *ybhC*, *yciA*, *menI*, and *yigI*) contributing to butyrate production. Lactate dehydrogenase (encoded by *ldh*) is the major contributor to lactate production and *mgsA* and *lldD* were related with lactate metabolism (37). Thus, these genes were used to the model as references to blast with the above high-quality genomes at protein level using BLASTP (v 2.4.0+) and hits with an e-value <1 × 10^−5^, percent identity ≥80%, and alignment length ≥50 AA were considered positive.

The genomes were also subjected to GhostKOALA (76) for annotations with KEGG, subsequent filtering based on the above genes was performed with manual inspections.

### Statistical analysis

The R “Stats” and “Vegan” packages were used to perform statistical analysis. PERMANOVA was performed to test whether the gut microbiota structure was significantly different by using the method implemented in the R “Vegan” package, and the *P* values were obtained with 999 permutations. The Mann-Whitney test and paired sample Wilcoxon signed rank test were used for univariate statistical analysis. The microbial correlation network was constructed using SparCC (sparse correlations for compositional data) (77) and correlated genus pairs were selected if the absolute value of sparse correlation |*r*| > 0.3 and *P* < 0.01.

To identify the bacterial taxa that can characterize ages of giant pandas or culturing days, we used the random forest (RF) model in R (ntree = 1,000) with default parameters (78). Lists of taxa ranked by RF in order of feature importance were determined over 100 iterations. The number of marker taxa was identified using 10-fold cross-validation implemented with the function in the R package “randomForest” with five repeats. The number of classes against cross-validation error curve became stable was used to estimate the importance of ASVs for explaining age and culturing day groups and to validate the RF analysis outcome. Spearman or Pearson rank correlation coefficients were used to assess the correlation between taxonomic relative abundance with increasing concentrations of each SCFAs and the level of significance was kept at the default of *P* < 0.05.

The online tool ImageGP (http://www.ehbio.com/ImageGP/) (79) was used for the data visualization, and the code of Fig. 7 was from https://github.com/iMetaScience/iMetaPlot/tree/main/221027Circlize (80).

## Data Availability

The raw sequencing reads from this study have been deposited into CNGB Sequence Archive (CNSA) of China National GeneBank DataBase (CNGBdb) with accession number CNP0005338.

## SUPPLEMENTAL MATERIAL

Supplemental material msystems.00520-24-s0001.pdf Supplemental figures and tables except Tables S3 and S4.

Table S3 msystems.00520-24-s0002.xlsx Information on samples of culture enrichment method and the statistical analysis of SCFAs among culture media.

Table S4 msystems.00520-24-s0003.xls Relative abundance and taxonomy of ASVs of culture-independent and culture enrichment methods.

ASM does not own the copyrights to Supplemental Material that may be linked to, or accessed through, an article. The authors have granted ASM a non-exclusive, world-wide license to publish the Supplemental Material files. Please contact the corresponding author directly for reuse.

## References

[1] Wang X, Yan Q, Xia X, Zhang Y, Li D, Wang C, Chen S, Hou R. 2013. Serotypes, virulence factors, and antimicrobial susceptibilities of vaginal and fecal isolates of Escherichia coli from giant pandas. Appl Environ Microbiol 79:5146–5150. doi:10.1128/AEM.01367-13 23793635 PMC3753950

[2] Zhao N, Li M, Luo J, Wang S, Liu S, Wang S, Lyu W, Chen L, Su W, Ding H, He H. 2017. Impacts of canine distemper virus infection on the giant panda population from the perspective of gut microbiota. Sci Rep 7:39954. doi:10.1038/srep39954 28051146 PMC5209704

[3] Zhao S, Li C, Zhu T, Jin L, Deng W, Zhao K, He Y, Li G, Xiong Y, Li T, Li B, Huang Y, Zhang H, Zou L. 2021. Diversity and composition of gut bacterial community in giant panda with anorexia. Curr Microbiol 78:1358–1366. doi:10.1007/s00284-021-02424-w 33646379

[4] Zou W, Li C, Yang X, Wang Y, Cheng G, Zeng J, Zhang X, Chen Y, Cai R, Huang Q, Feng L, Wang H, Li D, Zhang G, Chen Y, Zhang Z, Zhang H. 2018. Frequency of antimicrobial resistance and integron gene cassettes in Escherichia coli isolated from giant pandas (Ailuropoda melanoleuca) in China. Microb Pathog 116:173–179. doi:10.1016/j.micpath.2018.01.034 29414607

[5] Park MR, Ryu S, Maburutse BE, Oh NS, Kim SH, Oh S, Jeong SY, Jeong DY, Oh S, Kim Y. 2018. Probiotic Lactobacillus fermentum strain JDFM216 stimulates the longevity and immune response of Caenorhabditis elegans through a nuclear hormone receptor. Sci Rep 8:7441. doi:10.1038/s41598-018-25333-8 29748542 PMC5945636

[6] Li M, Wu X, Guo Z, Gao R, Ni Z, Cui H, Zong M, Van Bockstaele F, Lou W. 2023. Lactiplantibacillus plantarum enables blood urate control in mice through degradation of nucleosides in gastrointestinal tract. Microbiome 11:153. doi:10.1186/s40168-023-01605-y 37468996 PMC10354915

[7] Sugimura N, Li Q, Chu ESH, Lau HCH, Fong W, Liu W, Liang C, Nakatsu G, Su ACY, Coker OO, Wu WKK, Chan FKL, Yu J. 2021. Lactobacillus gallinarum modulates the gut microbiota and produces anti-cancer metabolites to protect against colorectal tumourigenesis. Gut 71:2011–2021. doi:10.1136/gutjnl-2020-323951 34937766 PMC9484392

[8] Bender MJ, McPherson AC, Phelps CM, Pandey SP, Laughlin CR, Shapira JH, Medina Sanchez L, Rana M, Richie TG, Mims TS, Gocher-Demske AM, Cervantes-Barragan L, Mullett SJ, Gelhaus SL, Bruno TC, Cannon N, McCulloch JA, Vignali DAA, Hinterleitner R, Joglekar AV, Pierre JF, Lee STM, Davar D, Zarour HM, Meisel M. 2023. Dietary tryptophan metabolite released by intratumoral Lactobacillus reuteri facilitates immune checkpoint inhibitor treatment. Cell 186:1846–1862. doi:10.1016/j.cell.2023.03.011 37028428 PMC10148916

[9] Archambaud C, Nahori MA, Soubigou G, Bécavin C, Laval L, Lechat P, Smokvina T, Langella P, Lecuit M, Cossart P. 2012. Impact of lactobacilli on orally acquired listeriosis. Proc Natl Acad Sci U S A 109:16684–16689. doi:10.1073/pnas.1212809109 23012479 PMC3478606

[10] Yu Q, Yuan L, Deng J, Yang Q. 2015. Lactobacillus protects the integrity of intestinal epithelial barrier damaged by pathogenic bacteria. Front Cell Infect Microbiol 5:26. doi:10.3389/fcimb.2015.00026 25859435 PMC4373387

[11] Liu Q, Ni X, Wang Q, Peng Z, Niu L, Wang H, Zhou Y, Sun H, Pan K, Jing B, Zeng D. 2017. Lactobacillus plantarum BSGP201683 isolated from giant panda feces attenuated inflammation and improved gut microflora in mice challenged with enterotoxigenic Escherichia coli. Front Microbiol 8:1885. doi:10.3389/fmicb.2017.01885 29018435 PMC5623042

[12] Yang HP, Qing-Yi MA, Gao R. 2015. Culture characteristics and drug sensitivity test of two Bifidobacterium strains in panda. Prog Vet Med 6:1007–5038. doi:10.16437/j.cnki.1007-5038.2015.06.040

[13] Zhou Y, Ni X, Duan L, Niu L, Liu Q, Zeng Y, Wang Q, Wang J, Khalique A, Pan K, Jing B, Zeng D. 2021. Lactobacillus plantarum BSGP201683 improves the intestinal barrier of giant panda microbiota-associated mouse infected by enterotoxigenic Escherichia coli K88. Probiotics Antimicro Prot 13:664–676. doi:10.1007/s12602-020-09722-y 33190214

[14] Zhou Y, Duan L, Zeng Y, Niu L, Pu Y, Jacobs JP, Chang C, Wang J, Khalique A, Pan K, Fang J, Jing B, Zeng D, Ni X. 2021. The panda-derived Lactobacillus plantarum G201683 alleviates the inflammatory response in DSS-induced panda microbiota-associated mice. Front Immunol 12:747045. doi:10.3389/fimmu.2021.747045 34956180 PMC8692892

[15] Liu Q, Ni X, Wang Q, Peng Z, Niu L, Xie M, Lin Y, Zhou Y, Sun H, Pan K, Jing B, Zeng D. 2019. Investigation of lactic acid bacteria isolated from giant panda feces for potential probiotics in vitro. Probiotics Antimicrob Proteins 11:85–91. doi:10.1007/s12602-017-9381-8 29353415

[16] Wang LM, Zhang WP, Wang J, Xie JJ, Zhou JL. 2019. Isolation, identification and analysis of some biological characteristics of Lactobacillus Salivarius from giant Panda. Chin J Wildl 40:537–546.

[17] Du X, Dai F, Yao F, Tan M, Pan Q. 2018. Genome sequence of Weissella cibaria M2, a potential probiotic strain isolated from the feces of a giant panda. Microbiol Resour Announc 7:e01121-18. doi:10.1128/MRA.01121-18 30533633 PMC6256656

[18] Xiong L, Ni X, Niu L, Zhou Y, Wang Q, Khalique A, Liu Q, Zeng Y, Shu G, Pan K, Jing B, Zeng D. 2019. Isolation and preliminary screening of a Weissella confusa strain from giant panda (Ailuropoda melanoleuca). Probiotics Antimicrob Proteins 11:535–544. doi:10.1007/s12602-018-9402-2 29654473

[19] Lagier JC, Dubourg G, Million M, Cadoret F, Bilen M, Fenollar F, Levasseur A, Rolain JM, Fournier PE, Raoult D. 2018. Culturing the human microbiota and culturomics. Nat Rev Microbiol 16:540–550. doi:10.1038/s41579-018-0041-0 29937540

[20] Pan W, Kang Y. 2018. Role of the microbiota in cancer growth and necrosis: the challenges and opportunities of bacteriotherapy for cancer and its complications. Rev Med Microbiol 29:20–23. doi:10.1097/MRM.0000000000000120

[21] Ihekweazu FD, Fofanova TY, Queliza K, Nagy-Szakal D, Stewart CJ, Engevik MA, Hulten KG, Tatevian N, Graham DY, Versalovic J, Petrosino JF, Kellermayer R. 2019. Bacteroides ovatus ATCC 8483 monotherapy is superior to traditional fecal transplant and multi-strain bacteriotherapy in a murine colitis model. Gut Microbes 10:504–520. doi:10.1080/19490976.2018.1560753 30663928 PMC6748610

[22] Lagier J-C, Hugon P, Khelaifia S, Fournier P-E, La Scola B, Raoult D. 2015. The rebirth of culture in microbiology through the example of culturomics to study human gut microbiota. Clin Microbiol Rev 28:237–264. doi:10.1128/CMR.00014-14 25567229 PMC4284300

[23] Lau JT, Whelan FJ, Herath I, Lee CH, Collins SM, Bercik P, Surette MG. 2016. Capturing the diversity of the human gut microbiota through culture-enriched molecular profiling. Genome Med 8:72. doi:10.1186/s13073-016-0327-7 27363992 PMC4929786

[24] Raymond F, Boissinot M, Ouameur AA, Déraspe M, Plante P-L, Kpanou SR, Bérubé È, Huletsky A, Roy PH, Ouellette M, Bergeron MG, Corbeil J. 2019. Culture-enriched human gut microbiomes reveal core and accessory resistance genes. Microbiome 7:56. doi:10.1186/s40168-019-0669-7 30953542 PMC6451232

[25] Ito T, Sekizuka T, Kishi N, Yamashita A, Kuroda M. 2019. Conventional culture methods with commercially available media unveil the presence of novel culturable bacteria. Gut Microbes 10:77–91. doi:10.1080/19490976.2018.1491265 30118379 PMC6363062

[26] Wang X, Howe S, Wei X, Deng F, Tsai T, Chai J, Xiao Y, Yang H, Maxwell CV, Li Y, Zhao J. 2021. Comprehensive cultivation of the swine gut microbiome reveals high bacterial diversity and guides bacterial isolation in pigs. mSystems 6:e0047721. doi:10.1128/mSystems.00477-21 34282935 PMC8407297

[27] Xiao S, Jiang S, Qian D, Duan J. 2020. Modulation of microbially derived short-chain fatty acids on intestinal homeostasis, metabolism, and neuropsychiatric disorder. Appl Microbiol Biotechnol 104:589–601. doi:10.1007/s00253-019-10312-4 31865438

[28] Luu M, Visekruna A. 2021. Microbial metabolites: novel therapeutic tools for boosting cancer therapies. Trends Cell Biol 31:873–875. doi:10.1016/j.tcb.2021.08.005 34538658

[29] Heilig H, Zoetendal EG, Vaughan EE, Marteau P, Akkermans ADL, de Vos WM. 2002. Molecular diversity of Lactobacillus spp. and other lactic acid bacteria in the human intestine as determined by specific amplification of 16S ribosomal DNA. Appl Environ Microbiol 68:114–123. doi:10.1128/AEM.68.1.114-123.2002 11772617 PMC126540

[30] Zhang W, Liu W, Hou R, Zhang L, Schmitz-Esser S, Sun H, Xie J, Zhang Y, Wang C, Li L, Yue B, Huang H, Wang H, Shen F, Zhang Z. 2018. Age-associated microbiome shows the giant panda lives on hemicelluloses, not on cellulose. ISME J 12:1319–1328. doi:10.1038/s41396-018-0051-y 29391488 PMC5931968

[31] Xue Z, Zhang W, Wang L, Hou R, Zhang M, Fei L, Zhang X, Huang H, Bridgewater LC, Jiang Y, Jiang C, Zhao L, Pang X, Zhang Z. 2015. The bamboo-eating giant panda harbors a carnivore-like gut microbiota, with excessive seasonal variations. mBio 6:e00022-15. doi:10.1128/mBio.00022-15 25991678 PMC4442137

[32] Bolyen E, Rideout JR, Dillon MR, Bokulich NA, Abnet CC, Al-Ghalith GA, Alexander H, Alm EJ, Arumugam M, Asnicar F, et al.. 2019. Reproducible, interactive, scalable and extensible microbiome data science using QIIME 2. Nat Biotechnol 37:852–857. doi:10.1038/s41587-019-0209-9 31341288 PMC7015180

[33] Bennett GM, Moran NA. 2013. Small, smaller, smallest: the origins and evolution of ancient dual symbioses in a phloem-feeding insect. Genome Biol Evol 5:1675–1688. doi:10.1093/gbe/evt118 23918810 PMC3787670

[34] Liu B, Zheng DD, Zhou SY, Chen LH, Yang J. 2022. VFDB 2022: a general classification scheme for bacterial virulence factors. Nucleic Acids Res 50:D912–D917. doi:10.1093/nar/gkab1107 34850947 PMC8728188

[35] Louis P, Hold GL, Flint HJ. 2014. The gut microbiota, bacterial metabolites and colorectal cancer. Nat Rev Microbiol 12:661–672. doi:10.1038/nrmicro3344 25198138

[36] Louis P, Duncan SH, McCrae SI, Millar J, Jackson MS, Flint HJ. 2004. Restricted distribution of the butyrate kinase pathway among butyrate-producing bacteria from the human colon. J Bacteriol 186:2099–2106. doi:10.1128/JB.186.7.2099-2106.2004 15028695 PMC374397

[37] Zhao C, Dong H, Zhang Y, Li Y. 2019. Discovery of potential genes contributing to the biosynthesis of short-chain fatty acids and lactate in gut microbiota from systematic investigation in E. coli. NPJ Biofilms Microbiomes 5:19. doi:10.1038/s41522-019-0092-7 31312512 PMC6626047

[38] Schaller GB, Hu J, Pan W, Zhu J. 1985. The giant pandas of wolong. University of Chicago Press, Chicago, USA.

[39] Rybiski Tarou L, Williams J, Powell DM, Tabet R, Allen M. 2005. Behavioral preferences for bamboo in a pair of captive giant pandas (Ailuropoda melanoleuca). Zoo Biol 24:177–183. doi:10.1002/zoo.20038

[40] Hansen RL, Carr MM, Apanavicius CJ, Jiang P, Bissell HA, Gocinski BL, Maury F, Himmelreich M, Beard S, Ouellette JR, Kouba AJ. 2010. Seasonal shifts in giant panda feeding behavior: relationships to bamboo plant part consumption. Zoo Biol 29:470–483. doi:10.1002/zoo.20280 19862794

[41] Williams CL, Willard S, Kouba A, Sparks D, Holmes W, Falcone J, Williams CH, Brown A. 2013. Dietary shifts affect the gastrointestinal microflora of the giant panda (Ailuropoda melanoleuca). J Anim Physiol Anim Nutr (Berl) 97:577–585. doi:10.1111/j.1439-0396.2012.01299.x 22524500

[42] Williams CL, Dill-McFarland KA, Vandewege MW, Sparks DL, Willard ST, Kouba AJ, Suen G, Brown AE. 2016. Dietary shifts may trigger dysbiosis and mucous stools in giant pandas (Ailuropoda melanoleuca). Front Microbiol 7:661. doi:10.3389/fmicb.2016.00661 27199976 PMC4858621

[43] Liu F, Li R, Zhong Y, Liu X, Deng W, Huang X, Price M, Li J. 2023. Age-related alterations in metabolome and microbiome provide insights in dietary transition in giant pandas. mSystems 8:e0025223. doi:10.1128/msystems.00252-23 37273228 PMC10308887

[44] Adamberg S, Tomson K, Vija H, Puurand M, Kabanova N, Visnapuu T, Jõgi E, Alamäe T, Adamberg K. 2014. Degradation of fructans and production of propionic acid by bacteroides thetaiotaomicron are enhanced by the shortage of amino acids. Front Nutr 1:21. doi:10.3389/fnut.2014.00021 25988123 PMC4428435

[45] Li D, Li Y, Dai W, Wang H, Qiu C, Feng S, Zhou Q, Wang W, Feng X, Yao K, Liu Y, Yang Y, Yang Z, Xu X, Li S, Wei J, Zhou K. 2019. Intestinal Bacteroides sp. imbalance associated with the occurrence of childhood undernutrition in China. Front Microbiol 10:2635. doi:10.3389/fmicb.2019.02635 31849851 PMC6895006

[46] Kircher B, Woltemate S, Gutzki F, Schlüter D, Geffers R, Bähre H, Vital M. 2022. Predicting butyrate- and propionate-forming bacteria of gut microbiota from sequencing data. Gut Microbes 14:2149019. doi:10.1080/19490976.2022.2149019 36416760 PMC9704393

[47] Sun J, Li H, Jin Y, Yu J, Mao S, Su KP, Ling Z, Liu J. 2021. Probiotic Clostridium butyricum ameliorated motor deficits in a mouse model of Parkinson's disease via gut microbiota-GLP-1 pathway. Brain Behav Immun 91:703–715. doi:10.1016/j.bbi.2020.10.014 33148438

[48] Zhang L, Zhang L, Zhan X, Zeng X, Zhou L, Cao G, Chen A, Yang C. 2016. Effects of dietary supplementation of probiotic, Clostridium butyricum, on growth performance, immune response, intestinal barrier function, and digestive enzyme activity in broiler chickens challenged with Escherichia coli K88. J Anim Sci Biotechnol 7:3. doi:10.1186/s40104-016-0061-4 26819705 PMC4728939

[49] Goodman AL, Kallstrom G, Faith JJ, Reyes A, Moore A, Dantas G, Gordon JI. 2011. Extensive personal human gut microbiota culture collections characterized and manipulated in gnotobiotic mice. Proc Natl Acad Sci U S A 108:6252–6257. doi:10.1073/pnas.1102938108 21436049 PMC3076821

[50] Kozich JJ, Westcott SL, Baxter NT, Highlander SK, Schloss PD. 2013. Development of a dual-index sequencing strategy and curation pipeline for analyzing amplicon sequence data on the MiSeq Illumina sequencing platform. Appl Environ Microbiol 79:5112–5120. doi:10.1128/AEM.01043-13 23793624 PMC3753973

[51] Magoč T, Salzberg SL. 2011. FLASH: fast length adjustment of short reads to improve genome assemblies. Bioinformatics 27:2957–2963. doi:10.1093/bioinformatics/btr507 21903629 PMC3198573

[52] Pruesse E, Quast C, Knittel K, Fuchs BM, Ludwig W, Peplies J, Glöckner FO. 2007. SILVA: a comprehensive online resource for quality checked and aligned ribosomal RNA sequence data compatible with ARB. Nucleic Acids Res 35:7188–7196. doi:10.1093/nar/gkm864 17947321 PMC2175337

[53] Dhariwal A, Chong J, Habib S, King IL, Agellon LB, Xia J. 2017. MicrobiomeAnalyst: a web-based tool for comprehensive statistical, visual and meta-analysis of microbiome data. Nucleic Acids Res 45:W180–W188. doi:10.1093/nar/gkx295 28449106 PMC5570177

[54] Zhang WP, Zheng LJ, Xie JJ, Hou R, Wang HR, Zhang ZH, Huang H, Chen P, Zhang L, Shen FJ, Liu JW. 2022. An online detection method of short chain fatty acids in giant panda feces based on gas chromatography. Patent No: 202110037455. (In Chinese).

[55] Kolmogorov M, Yuan J, Lin Y, Pevzner PA. 2019. Assembly of long, error-prone reads using repeat graphs. Nat Biotechnol 37:540–546. doi:10.1038/s41587-019-0072-8 30936562

[56] De Coster W, D’Hert S, Schultz DT, Cruts M, Van Broeckhoven C. 2018. NanoPack: visualizing and processing long-read sequencing data. Bioinformatics 34:2666–2669. doi:10.1093/bioinformatics/bty149 29547981 PMC6061794

[57] Kang DD, Li F, Kirton E, Thomas A, Egan R, An H, Wang Z. 2019. MetaBAT 2: an adaptive binning algorithm for robust and efficient genome reconstruction from metagenome assemblies. PeerJ 7:e7359. doi:10.7717/peerj.7359 31388474 PMC6662567

[58] Alneberg J, Bjarnason BS, de Bruijn I, Schirmer M, Quick J, Ijaz UZ, Lahti L, Loman NJ, Andersson AF, Quince C. 2014. Binning metagenomic contigs by coverage and composition. Nat Methods 11:1144–1146. doi:10.1038/nmeth.3103 25218180

[59] Wu YW, Tang YH, Tringe SG, Simmons BA, Singer SW. 2014. MaxBin: an automated binning method to recover individual genomes from metagenomes using an expectation-maximization algorithm. Microbiome 2:26. doi:10.1186/2049-2618-2-26 25136443 PMC4129434

[60] Langmead B, Salzberg SL. 2012. Fast gapped-read alignment with Bowtie 2. Nat Methods 9:357–359. doi:10.1038/nmeth.1923 22388286 PMC3322381

[61] Li H. 2018. Minimap2: pairwise alignment for nucleotide sequences. Bioinformatics 34:3094–3100. doi:10.1093/bioinformatics/bty191 29750242 PMC6137996

[62] Uritskiy GV, DiRuggiero J, Taylor J. 2018. MetaWRAP -- a flexible pipeline for genome resolved metagenomic data analysis. Microbiome 6:158. doi:10.1186/s40168-018-0541-1 30219103 PMC6138922

[63] Parks DH, Imelfort M, Skennerton CT, Hugenholtz P, Tyson GW. 2015. CheckM: assessing the quality of microbial genomes recovered from isolates, single cells, and metagenomes. Genome Res 25:1043–1055. doi:10.1101/gr.186072.114 25977477 PMC4484387

[64] Hyatt D, Chen G-L, Locascio PF, Land ML, Larimer FW, Hauser LJ. 2010. Prodigal: prokaryotic gene recognition and translation initiation site identification. BMC Bioinformatics 11:119. doi:10.1186/1471-2105-11-119 20211023 PMC2848648

[65] von Meijenfeldt FAB, Arkhipova K, Cambuy DD, Coutinho FH, Dutilh BE. 2019. Robust taxonomic classification of uncharted microbial sequences and bins with CAT and BAT. Genome Biol 20:217. doi:10.1186/s13059-019-1817-x 31640809 PMC6805573

[66] Buchfink B, Xie C, Huson DH. 2015. Fast and sensitive protein alignment using DIAMOND. Nat Methods 12:59–60. doi:10.1038/nmeth.3176 25402007

[67] Chaumeil PA, Mussig AJ, Hugenholtz P, Parks DH. 2019. GTDB-Tk: a toolkit to classify genomes with the genome taxonomy database. Bioinformatics 36:1925–1927. doi:10.1093/bioinformatics/btz848 31730192 PMC7703759

[68] Nguyen L-T, Schmidt HA, von Haeseler A, Minh BQ. 2015. IQ-TREE: a fast and effective stochastic algorithm for estimating maximum-likelihood phylogenies. Mol Biol Evol 32:268–274. doi:10.1093/molbev/msu300 25371430 PMC4271533

[69] Letunic I, Bork P. 2021. Interactive tree of life (iTOL) V5: an online tool for phylogenetic tree display and annotation. Nucleic Acids Res 49:W293–W296. doi:10.1093/nar/gkab301 33885785 PMC8265157

[70] Richter M, Rosselló-Móra R, Oliver Glöckner F, Peplies J. 2016. JSpeciesWS: a web server for prokaryotic species circumscription based on pairwise genome comparison. Bioinformatics 32:929–931. doi:10.1093/bioinformatics/btv681 26576653 PMC5939971

[71] Meier-Kolthoff JP, Göker M. 2019. TYGS is an automated high-throughput platform for state-of-the-art genome-based taxonomy. Nat Commun 10:2182. doi:10.1038/s41467-019-10210-3 31097708 PMC6522516

[72] Meier-Kolthoff JP, Carbasse JS, Peinado-Olarte RL, Göker M. 2022. TYGS and LPSN: a database tandem for fast and reliable genome-based classification and nomenclature of prokaryotes. Nucleic Acids Res 50:D801–D807. doi:10.1093/nar/gkab902 34634793 PMC8728197

[73] Alcock BP, Raphenya AR, Lau TTY, Tsang KK, Bouchard M, Edalatmand A, Huynh W, Nguyen A-LV, Cheng AA, Liu S, et al.. 2020. CARD 2020: antibiotic resistome surveillance with the comprehensive antibiotic resistance database. Nucleic Acids Res 48:D517–D525. doi:10.1093/nar/gkz935 31665441 PMC7145624

[74] Vital M, Karch A, Pieper DH. 2017. Colonic butyrate-producing communities in humans: an overview using omics data. mSystems 2:e00130-17. doi:10.1128/mSystems.00130-17 29238752 PMC5715108

[75] Zou Y, Xue W, Luo G, Deng Z, Qin P, Guo R, Sun H, Xia Y, Liang S, Dai Y, et al.. 2019. 1,520 reference genomes from cultivated human gut bacteria enable functional microbiome analyses. Nat Biotechnol 37:179–185. doi:10.1038/s41587-018-0008-8 30718868 PMC6784896

[76] Kanehisa M, Sato Y, Morishima K. 2016. BlastKOALA and GhostKOALA: KEGG tools for functional characterization of genome and metagenome sequences. J Mol Biol 428:726–731. doi:10.1016/j.jmb.2015.11.006 26585406

[77] Friedman J, Alm EJ. 2012. Inferring correlation networks from genomic survey data. PLoS Comput Biol 8:e1002687. doi:10.1371/journal.pcbi.1002687 23028285 PMC3447976

[78] Knights D, Kuczynski J, Koren O, Ley RE, Field D, Knight R, DeSantis TZ, Kelley ST. 2011. Supervised classification of microbiota mitigates mislabeling errors. ISME J 5:570–573. doi:10.1038/ismej.2010.148 20927137 PMC3105748

[79] Chen T, Liu Y, Huang L. 2022. ImageGP: an easy‐to‐use data visualization web server for scientific researchers. iMeta 1:e5. doi:10.1002/imt2.5 38867732 PMC10989750

[80] Zhu Y-X, Yang R, Wang X-Y, Wen T, Gong M-H, Shen Y, Xu J-Y, Zhao D-S, Du Y-Z. 2022. Gut microbiota composition in the sympatric and diet‐sharing Drosophila simulans and Dicranocephalus wallichii bowringi shaped largely by community assembly processes rather than regional species pool. iMeta 1:e57. doi:10.1002/imt2.57 38867909 PMC10989964

