# Supplementary material for: The giant panda gut harbors a high diversity of lactic acid bacteria revealed by a novel culturomics pipeline
Source: mSystems. 2024 Jun 26;9(7):e00520-24. doi: 10.1128/msystems.00520-24 (PMC11265448; doi:10.1128/msystems.00520-24)
Supplement: Supplemental material — Supplemental figures and tables except Tables S3 and S4. [file msystems.00520-24-s0001.pdf]

Supplementary data

The giant panda gut harbors a high diversity of lactic acid bacteria revealed by a novel culturomics pipeline

Wenping Zhang[1,*], Lijun Zheng[2], Junjin Xie[2], Xiaoyan Su[2], Mingchun Zhang[3], He Huang[2], Stephan Schmitz-Esser[4], Shizhang Du[1], Yu Yang[2], Jiqin Xie[2], Qinrong Zhang[2], Shuran Yu[2], Qiang Guo[2], Hairui Wang[2], Liang Zhang[2,3], Kong Yang[5,*], Rong Hou[2,*]

1 Key laboratory of monitoring biological diversity in Minshan mountain of national park of giant pandas, College of Life Science and Biotechnology, Mianyang Normal University, Mianyang 621000, Sichuan, China

2 Chengdu Research Base of Giant Panda Breeding, Chengdu 610081, Sichuan, China

3 China Conservation and Research Center for the Giant Panda, Chengdu, Sichuan 610081, China

4 Department of Animal Science, Iowa State University, Ames 50011, IA, USA

5 Institute of Qinghai-Tibetan Plateau, Southwest Minzu University, Chengdu 610041, Sichuan, China

*, To whom correspondence may be addressed**:**

Wenping Zhang: zhang_zoology@163.com

Kong Yang: lx-yk@163.com

Rong Hou: hourong2000@panda.org.cn

Includes:

Supplemental Figure Legends

Supplemental Table Legends

Supplemental Figures

Supplemental Tables

Supplemental Figure Legends

Figure S1: the pipeline of this study.

Figure S2: the relative abundance on genus level for culture-independent method. Every column denoted one sample.

Figure S3: The PCoA of weighted UniFrac analysis of culture-independent method. (R-squared = 0.15942; p-value < 0.001).

Figure S4: The Venn chart of all observed ASVs: all ASVs for every culture media. The details of culture media are shown in Table S2.

Figure S5: The PCoA of weighted (upper panel) and unweighted (lower panel) UniFrac analysis of culture-enriched samples based on age. YZ: cub (< 1 year old); WC: subadult (1-3 years old); CN: adult (> 4 years old).

Figure S6: The PCoA of unweighted (A) and weighted (B) UniFrac analysis and the PCA of Bray-Curtis distance (C) of culture-enriched samples based on culturing days.

Figure S7: Comparisons of SCFAs among culture media. The details of statistical analysis are shown in Table S3.

Figure S8: Phylogenetic distribution of 12 *Lactobacillus plantarum* genomes following the results of TYGS in culture-enriched cohort.

Figure S9: Phylogeny of Butyrate kinase (*Buk*). The tree was based on sequences from *Buk* using the Maximum Likelihood method based on the Poisson correction model in MEGA X. The percentage of trees in which the associated taxa clustered together is shown next to the branches. The number at the end of name for every strain showed the code of every copy of *Buk* in corresponding genome.

Figure S10: Phylogeny of Lactate dehydrogenase A (encoded by *ldhA*). The tree was based on sequences from *ldhA* using the Maximum Likelihood method based on the Poisson correction model in MEGA X. The percentage of trees in which the associated taxa clustered together is shown next to the branches. The number at the end of name for every strain showed the code of every copy of *ldhA* in corresponding genome.

Figure S11: Pearson's correlations of different ASVs in culture-enrichment with SCFAs. The number indicated the correlations (r^2). Here, only the positive correlation between the important ASVs and SCFAs are shown ($P < 0.05$).

Supplemental Table Legends

Table S1: the information of samples of culture-independent method in this study.

Table S2: the information of culture media in this study.

Table S3: the information of samples of culture-enrichment method and the statistical analysis of SCFAs among culture media. (a separate file)

Table S4: the relative abundance and taxonomy of ASVs of culture-independent and culture-enrichment methods. (a separate file)

Table S5: the ANI values (> 0.95) and closest species of genomes from JSpecies.

Table S6: the ANI values (< 95%) and taxonomy of genomes from JSpecies. Here showed the identification of TYGS and results of CheckM.

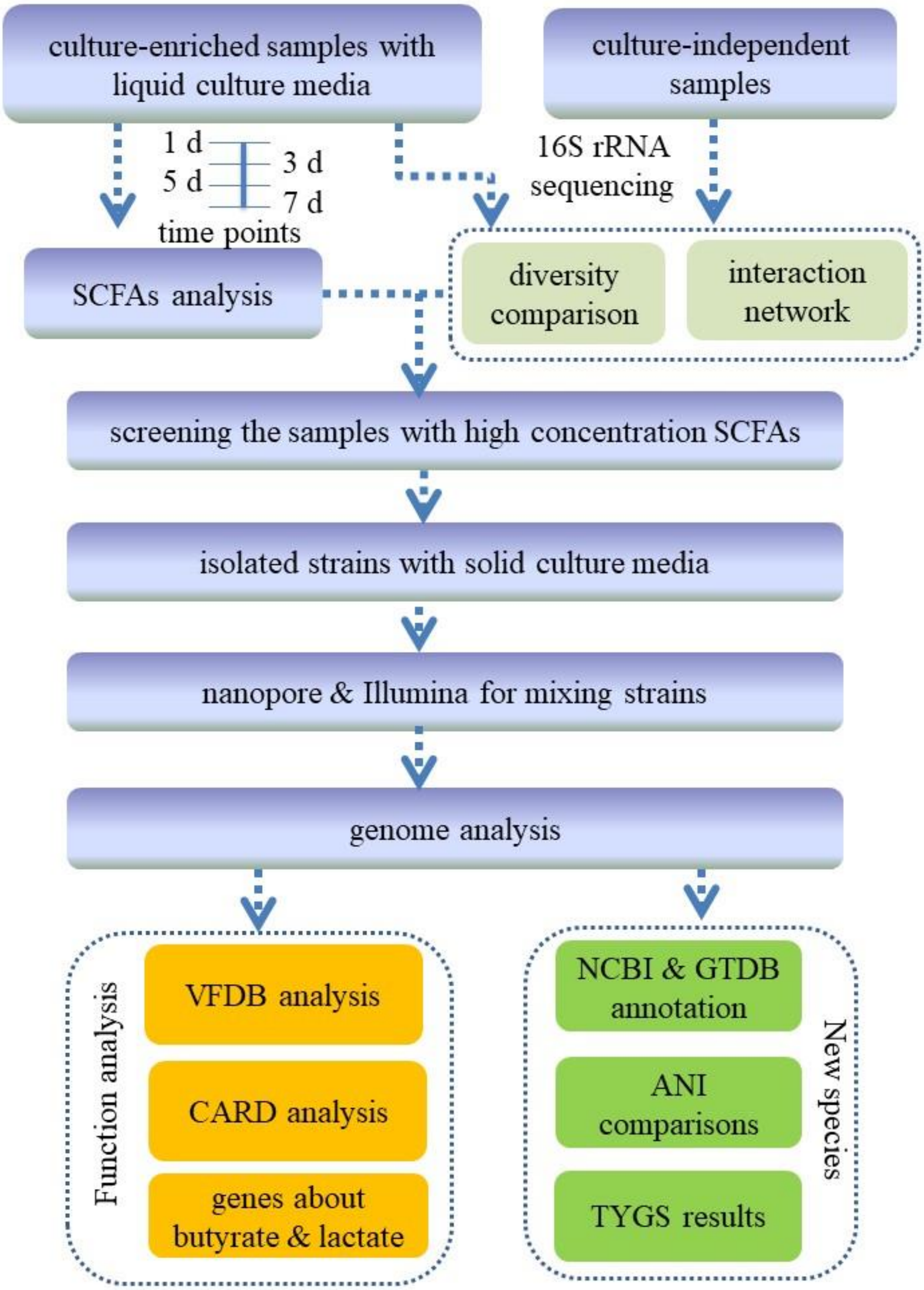

Figure S1: the pipeline of this study.

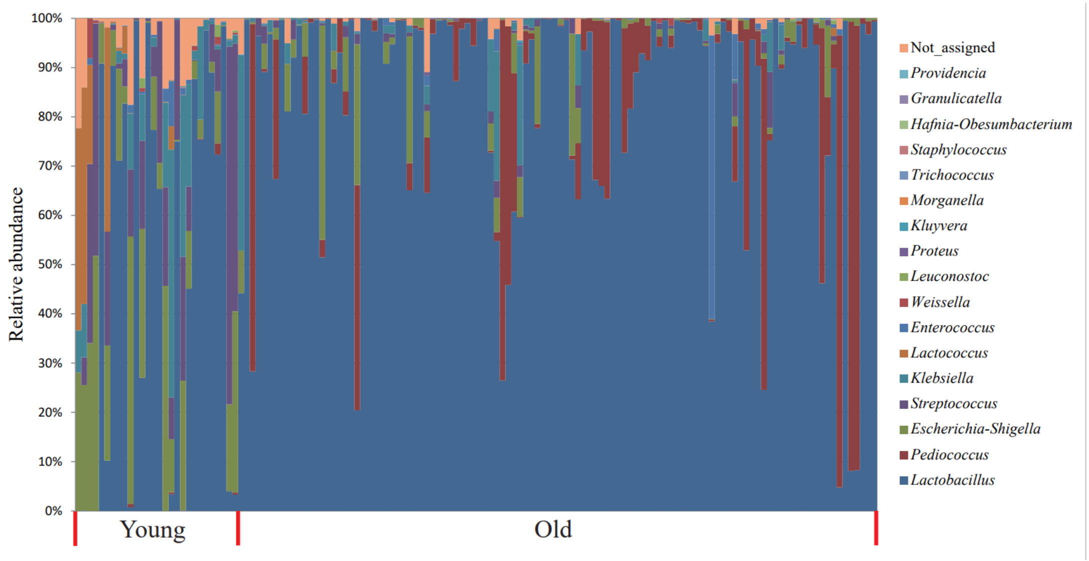

Figure S2: the relative abundance of genus level for culture-independent method. Every column denoted one sample.

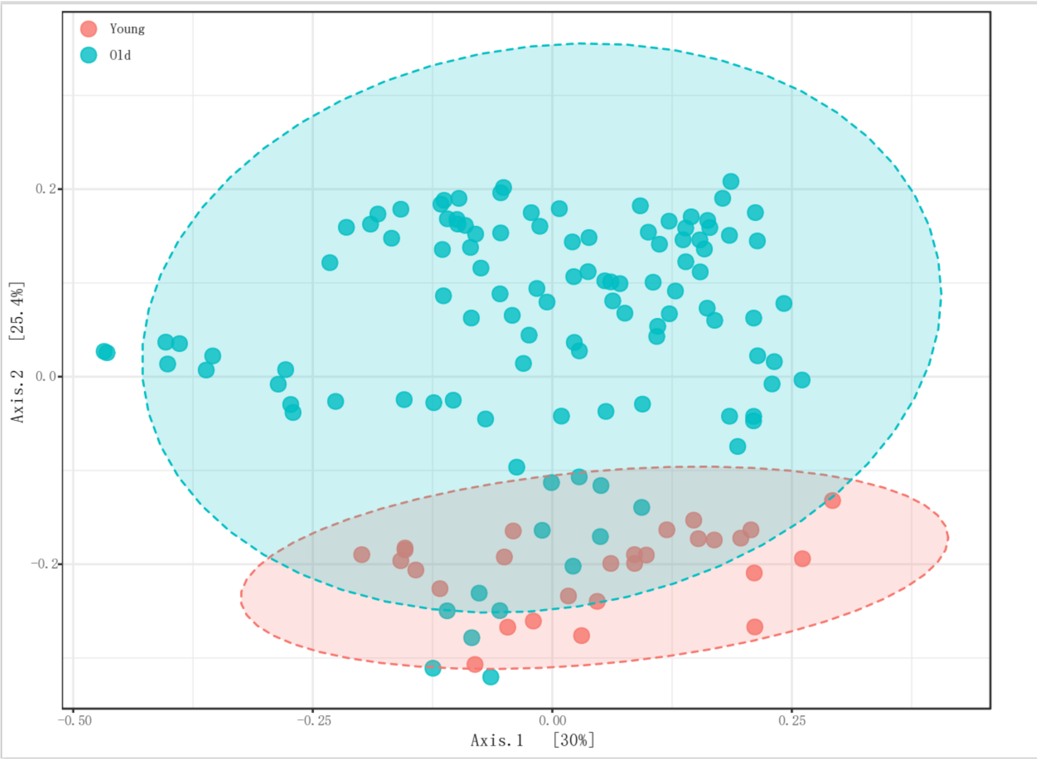

Figure S3: The PCoA of weighted UniFrac analysis of culture-independent method. (R-squared=0.15942; p-value < 0.001)

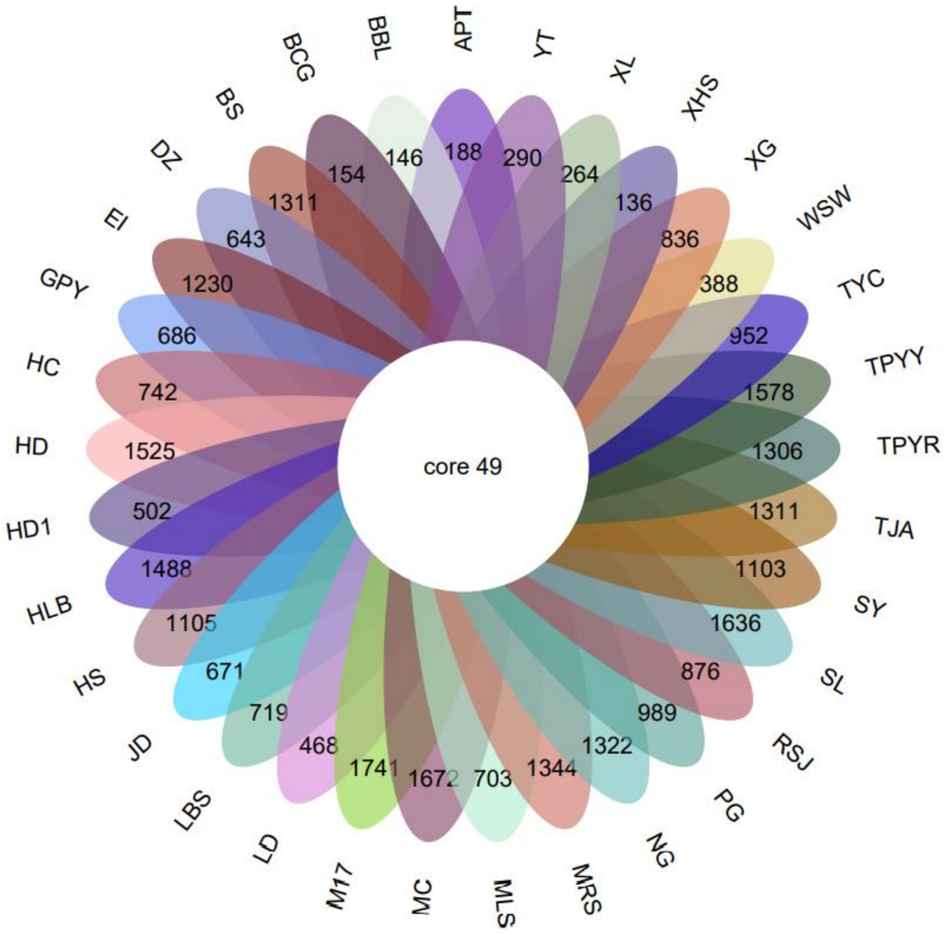

Figure S4: The venn chart of all observed ASVs: all ASVs for every culture media. The details of culture media showed in table S2.

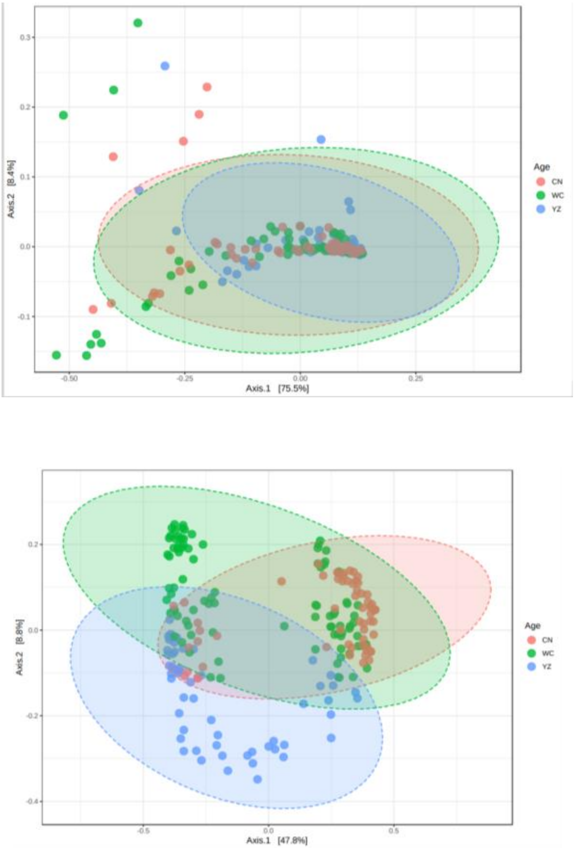

Figure S5: The PCoA of weighted (up) and unweighted (down) UniFrac analysis of culture-enriched samples based on age. YZ: cub (< 1 year old); WC: subadult (1-3 years old); CN: adult (> 4 years old).

(A)

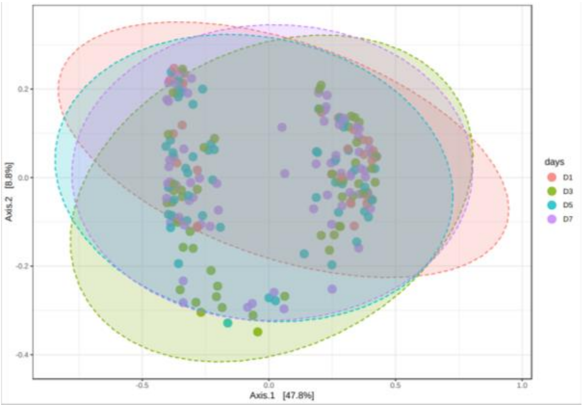

(B)

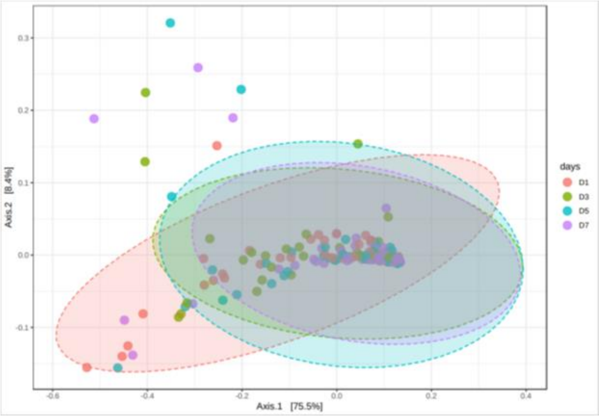

(C)

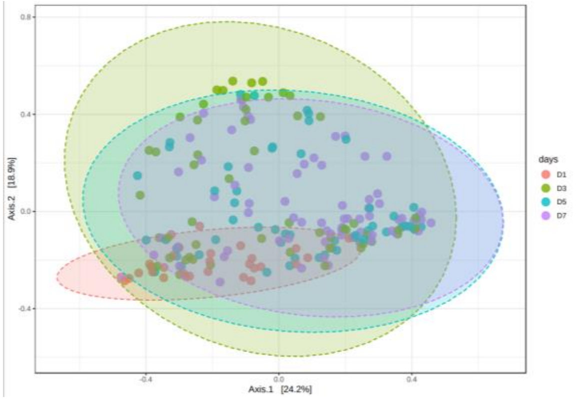

Figure S6: The PCoA of unweighted (A) and weighted (B) UniFrac analysis and the PCA of Bray-Curtis distance (C) of culture-enriched samples based on culturing days.

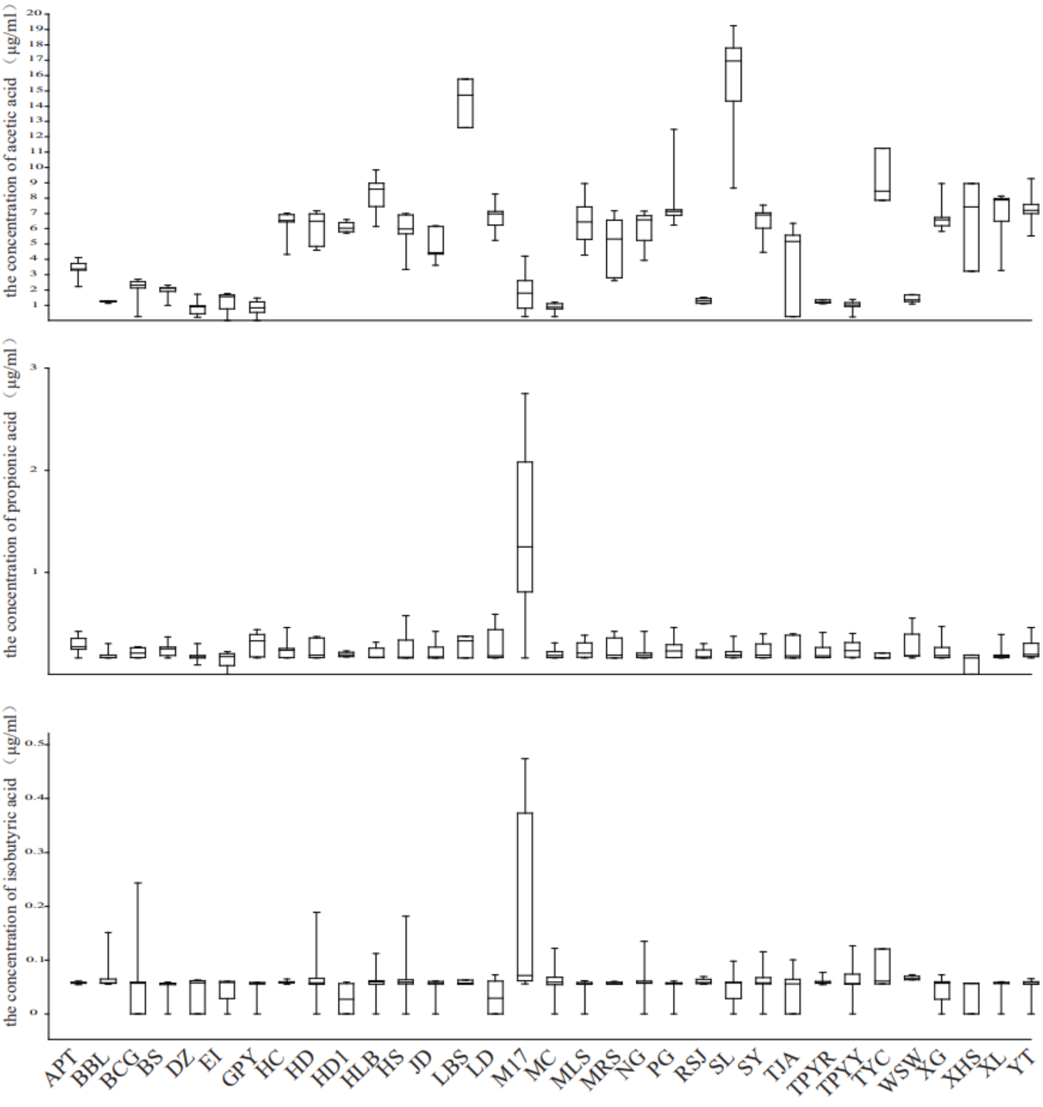

Figure S7: the comparisons of SCFAs among culture media. The details of statistic analysis showed in Table S3.

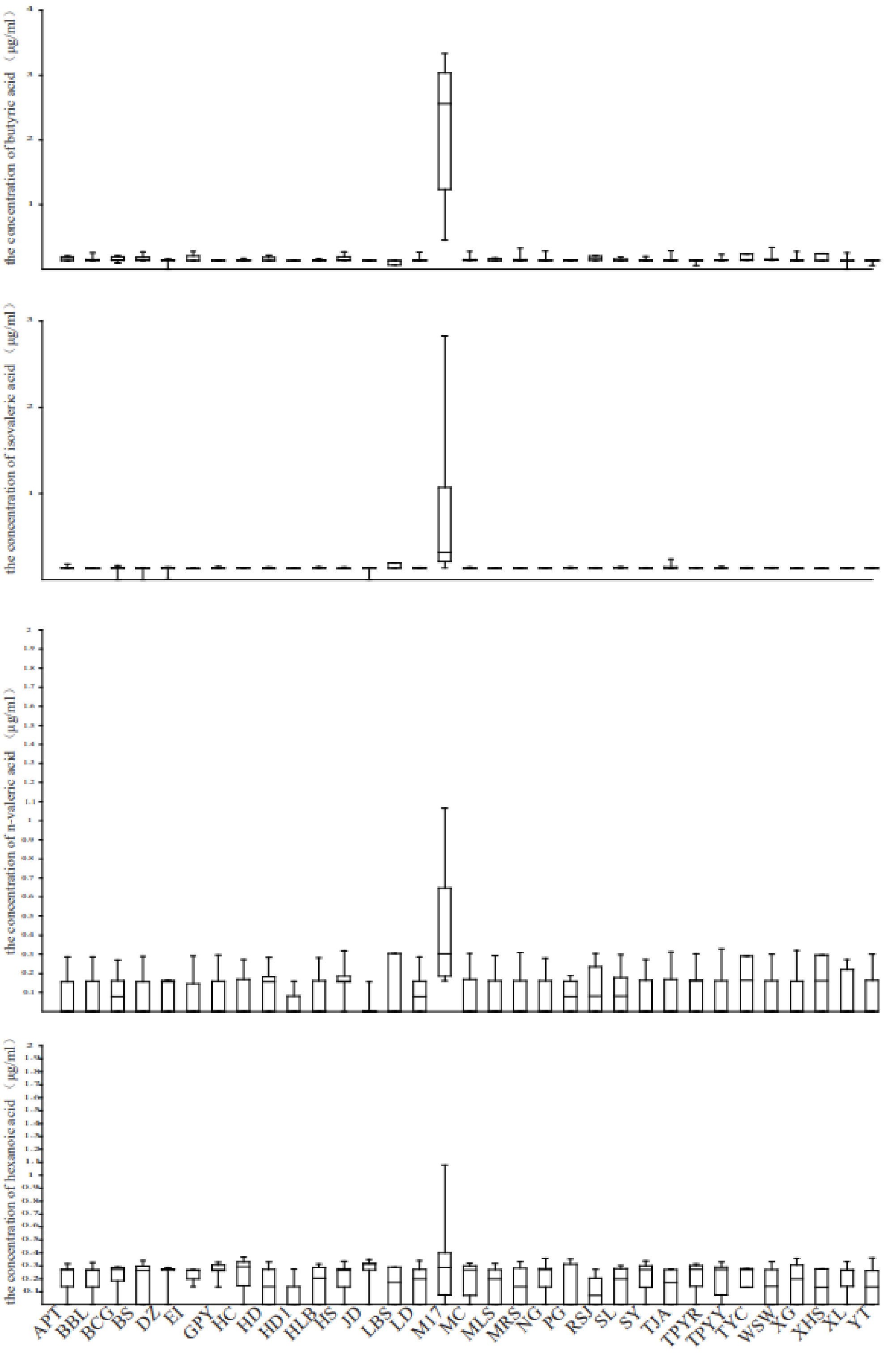

Continued Figure S7:

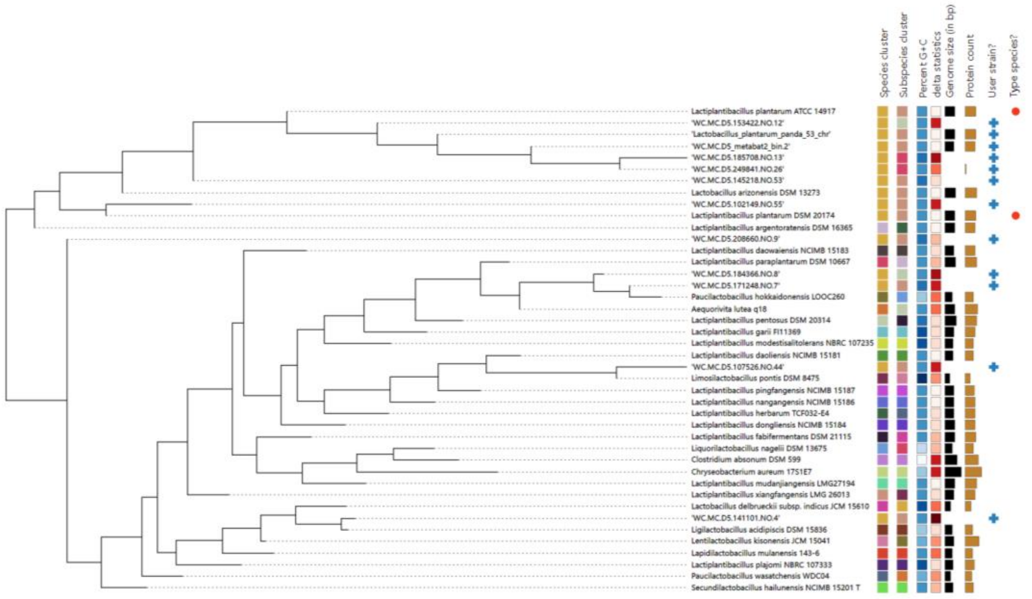

Figure S8：Phylogenetic distribution of 12 *Lactobacillus plantarum* genomes following the results of TYGS in culture-enriched cohort.

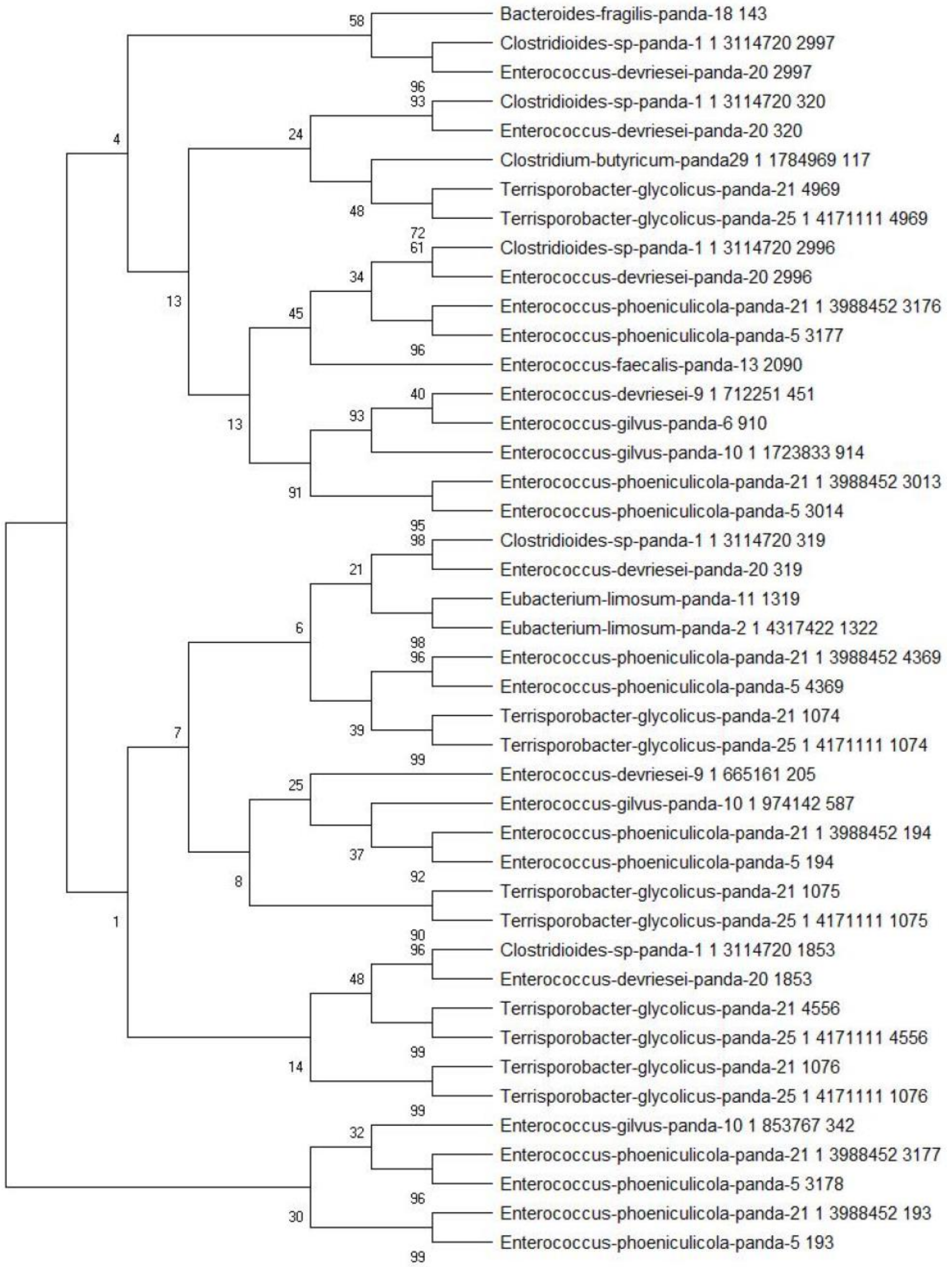

Figure S9: Phylogeny of Butyrate kinase (*Buk*). The tree was based on sequences from *Buk* using the Maximum Likelihood method based on the Poisson correction model in MEGA X. The percentage of trees in which the associated taxa clustered together is shown next to the branches. The number at the end of name for every strain showed the code of every copy of *Buk* in corresponding genome.

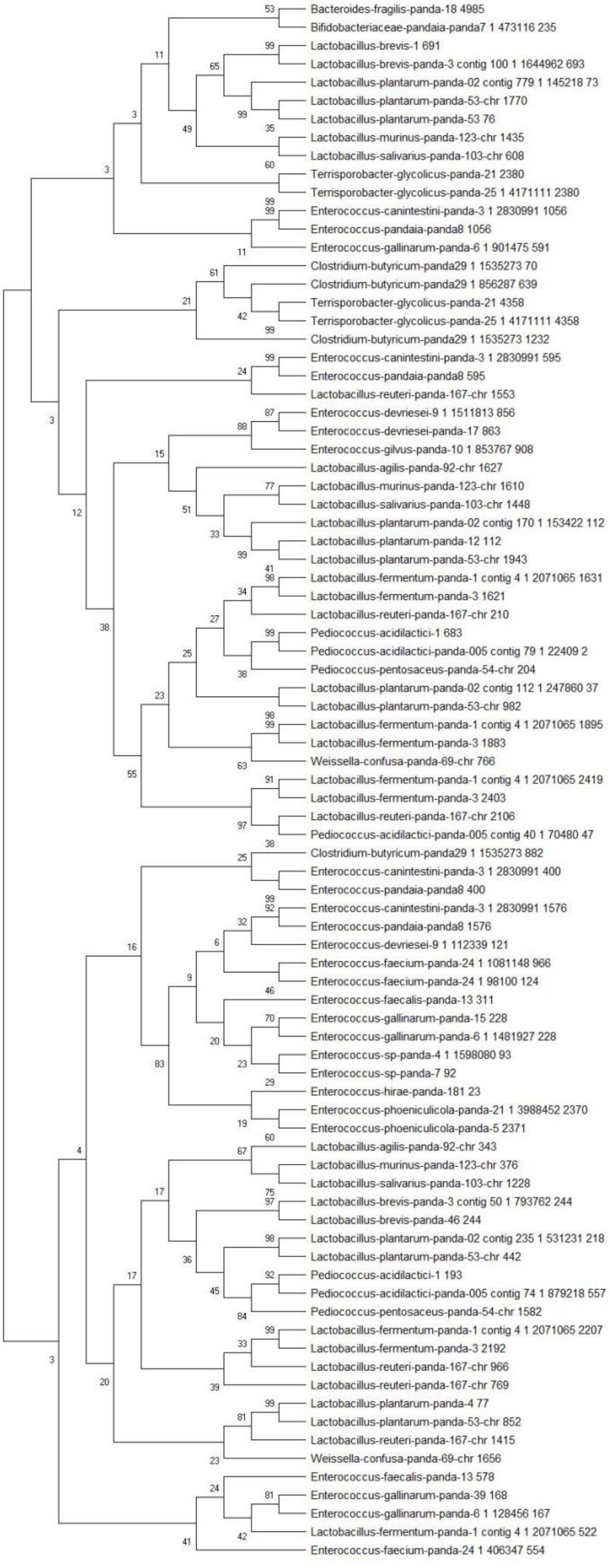
Bacteroides-fragilis-panda-18 4985
Bifidobacteriaceae-pandaia-panda7 1 473116 235
Lactobacillus-brevis-1 691
Lactobacillus-brevis-panda-3 contig 100 1 1644962 693
Lactobacillus-plantarum-panda-02 contig 779 1 145218 73
Lactobacillus-plantarum-panda-53-chr 1770
Lactobacillus-plantarum-panda-53 76
Lactobacillus-murinus-panda-123-chr 1435
Lactobacillus-salivarius-panda-103-chr 608
Terrisporobacter-glycolicus-panda-21 2380
Terrisporobacter-glycolicus-panda-25 1 4171111 2380
Enterococcus-canintestini-panda-3 1 2830991 1056
Enterococcus-pandaia-panda8 1056
Enterococcus-gallinarum-panda-6 1 901475 591
Clostridium-butyricum-panda29 1 1535273 70
Clostridium-butyricum-panda29 1 856287 639
Terrisporobacter-glycolicus-panda-21 4358
Terrisporobacter-glycolicus-panda-25 1 4171111 4358
Clostridium-butyricum-panda29 1 1535273 1232
Enterococcus-canintestini-panda-3 1 2830991 595
Enterococcus-pandaia-panda8 595
Lactobacillus-reuteri-panda-167-chr 1553
Enterococcus-devriesei-9 1 1511813 856
Enterococcus-devriesei-panda-17 863
Enterococcus-gilvus-panda-10 1 853767 908
Lactobacillus-agilis-panda-92-chr 1627
Lactobacillus-murinus-panda-123-chr 1610
Lactobacillus-salivarius-panda-103-chr 1448
Lactobacillus-plantarum-panda-02 contig 170 1 153422 112
Lactobacillus-plantarum-panda-12 112
Lactobacillus-plantarum-panda-53-chr 1943
Lactobacillus-fermentum-panda-1 contig 4 1 2071065 1631
Lactobacillus-fermentum-panda-3 1621
Lactobacillus-reuteri-panda-167-chr 210
Pediococcus-acidilactici-1 683
Pediococcus-acidilactici-panda-005 contig 79 1 22409 2
Pediococcus-pentosaceus-panda-54-chr 204
Lactobacillus-plantarum-panda-02 contig 112 1 247860 37
Lactobacillus-plantarum-panda-53-chr 982
Lactobacillus-fermentum-panda-1 contig 4 1 2071065 1895
Lactobacillus-fermentum-panda-3 1883
Weissella-confusa-panda-69-chr 766
Lactobacillus-fermentum-panda-1 contig 4 1 2071065 2419
Lactobacillus-fermentum-panda-3 2403
Lactobacillus-reuteri-panda-167-chr 2106
Pediococcus-acidilactici-panda-005 contig 40 1 70480 47
Clostridium-butyricum-panda29 1 1535273 882
Enterococcus-canintestini-panda-3 1 2830991 400
Enterococcus-pandaia-panda8 400
Enterococcus-canintestini-panda-3 1 2830991 1576
Enterococcus-pandaia-panda8 1576
Enterococcus-devriesei-9 1 112339 121
Enterococcus-faecium-panda-24 1 1081148 966
Enterococcus-faecium-panda-24 1 98100 124
Enterococcus-faecalis-panda-13 311
Enterococcus-gallinarum-panda-15 228
Enterococcus-gallinarum-panda-6 1 1481927 228
Enterococcus-sp-panda-4 1 1598080 93
Enterococcus-sp-panda-7 92
Enterococcus-hirae-panda-181 23
Enterococcus-phoeniculicola-panda-21 1 3988452 2370
Enterococcus-phoeniculicola-panda-5 2371
Lactobacillus-agilis-panda-92-chr 343
Lactobacillus-murinus-panda-123-chr 376
Lactobacillus-salivarius-panda-103-chr 1228
Lactobacillus-brevis-panda-3 contig 50 1 793762 244
Lactobacillus-brevis-panda-46 244
Lactobacillus-plantarum-panda-02 contig 235 1 531231 218
Lactobacillus-plantarum-panda-53-chr 442
Pediococcus-acidilactici-1 193
Pediococcus-acidilactici-panda-005 contig 74 1 879218 557
Pediococcus-pentosaceus-panda-54-chr 1582
Lactobacillus-fermentum-panda-1 contig 4 1 2071065 2207
Lactobacillus-fermentum-panda-3 2192
Lactobacillus-reuteri-panda-167-chr 966
Lactobacillus-reuteri-panda-167-chr 769
Lactobacillus-plantarum-panda-4 77
Lactobacillus-plantarum-panda-53-chr 852
Lactobacillus-reuteri-panda-167-chr 1415
Weissella-confusa-panda-69-chr 1656
Enterococcus-faecalis-panda-13 578
Enterococcus-gallinarum-panda-39 168
Enterococcus-gallinarum-panda-6 1 128456 167
Lactobacillus-fermentum-panda-1 contig 4 1 2071065 522
Enterococcus-faecium-panda-24 1 406347 554

Figure S10: Phylogeny of Lactate dehydrogenase A (encoded by *ldhA*). The tree was based on sequences from *ldhA* using the Maximum Likelihood method based on the Poisson correction model in MEGA X. The percentage of trees in which the associated taxa clustered together is shown next to the branches. The number at the end of name for every strain showed the code of every copy of *ldhA* in corresponding genome.

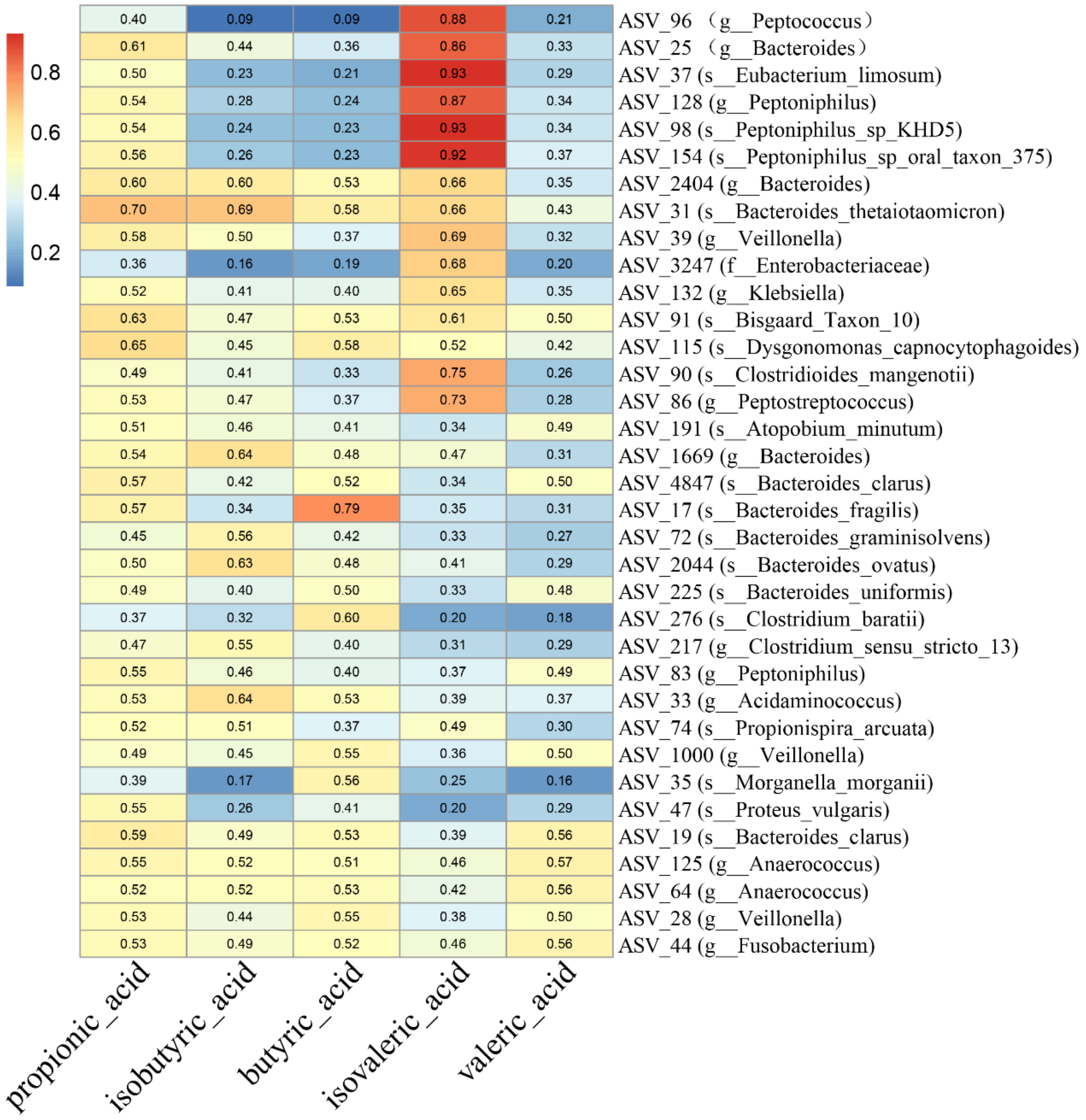

Figure S11: Pearson's correlations of different ASVs in culture-enrichment with SCFAs. The number indicated the correlations (r^2). Here only showed the positive correlation between the important ASVs and SCFAs ($P < 0.05$).

Table S1: The information of samples of culture-independent method in this study.

#NAME	Host Id	Days after birth	Age Id	Sequence Count	Observed ASVs	Shannon
12024.1A10SX12031	SX	123	Old	30711	55	1.479
12024.1A11JD12061	JD	126	Old	54574	73	1.924
12024.1A12JX12121	JX	132	Old	29424	89	2.706
12024.1A1KD1201	KD	162	Old	39184	40	1.115
12024.1A2KX1209	KX	170	Old	45244	55	1.244
12024.1A3YD1201	YD	128	Old	26991	58	1.627
12024.1A7ND1201	ND	116	Old	52126	73	1.982
12024.1A8NX1201	NX	116	Old	58360	75	1.839
12024.1B10SX1211	SX	131	Old	46510	77	2.321
12024.1B12YX1221	YX	148	Old	28256	55	1.732
12024.1B4YX1207	YX	134	Old	9886	40	1.411
12024.1B7ND12061	ND	121	Old	38957	76	2.077
12024.1B8NX12061	NX	121	Old	58020	79	2.534
12024.1B9QX1222	QX	97	Old	56991	53	1.312
12024.1C10SD1212	SD	132	Old	38315	83	2.444
12024.1C11SX12061	SX	126	Old	56760	64	1.731
12024.1C12JD12062	JD	136	Old	31141	75	2.338
12024.1C2KX1210	KX	171	Old	66600	73	1.317
12024.1C7QX1201	QX	76	Old	54469	68	2.218
12024.1D10ND0117	ND	163	Old	35966	72	2.724
12024.1D12QX0119	QX	125	Old	23519	66	2.379
12024.1D2ND0126	ND	172	Old	44326	66	2.535
12024.1D3SD0122	SD	173	Old	24837	70	3.048
12024.1D7QX0126	QX	114	Old	37957	59	2.049
12024.1D8QD01261	QD	132	Old	50994	66	2.495
12024.1E10QX0108	QX	114	Old	43826	62	2.208
12024.1E12QD0108	QD	114	Old	21576	54	2.378
12024.1E1KD0118	KD	210	Old	29560	63	2.172
12024.1E2ND0115	ND	161	Old	31747	97	3.251
12024.1E3QX0112	QX	118	Old	24640	67	2.375
12024.1E7QD0111	QD	117	Old	59582	56	2.144
12024.1E8QD01262	QD	132	Old	51275	58	1.920
12024.1F10QD12023	QD	77	Old	37561	77	2.591
12024.1F11NX1002	NX	56	Young	60179	57	1.316
12024.1F12ND1028	ND	82	Old	33371	71	1.736
12024.1F1ND1203	ND	118	Old	48300	77	2.440
12024.1F2KX1012	KX	112	Old	45115	76	2.573
12024.1F4SX12062	SX	126	Old	19981	51	2.141

12024.1F7NX12062	NX	121	Old	43657	72	2.439
12024.1G10YX1109	YX	106	Old	35879	68	1.963
12024.1G11JX12122	JX	132	Old	35094	82	2.148
12024.1G12QX1215	QX	90	Old	23353	48	1.266
12024.1G1NX1201	NX	116	Old	44024	77	2.242
12024.1G2MZ1009	MZ	96	Old	38027	60	2.466
12024.1G3JD1116	JD	106	Old	29754	66	1.391
12024.1G6MZ1110	MZ	128	Old	32912	80	1.836
12024.1G7YD11021	YD	99	Old	37886	61	1.248
12024.1G8YX1126	YX	123	Old	46266	80	2.223
12024.1H10ND0115	ND	161	Old	44203	76	2.523
12024.1H11JD1125	JD	115	Old	44979	70	2.601
12024.1H1QD1222	QD	97	Old	34534	51	1.345
12024.1H2YX1022	YX	88	Old	31917	75	2.810
12024.1H3YD11022	YD	99	Old	22564	57	1.233
12024.1H7NX1109	NX	94	Old	44636	70	2.192
12024.1H8ND1201	ND	116	Old	51821	84	2.320
12024.1H9KX11172	KX	148	Old	46834	87	2.204
12024.2A10SX1010	SX	69	Young	37505	50	2.003
12024.2A11JD1010	JD	69	Young	42852	57	1.916
12024.2A1KD1008	KD	108	Old	20714	81	2.575
12024.2A2KX1008	KX	108	Old	50248	62	2.322
12024.2A3YD1008	YD	68	Young	68444	58	2.309
12024.2A4YX	YX	68	Young	47761	41	0.936
12024.2A5QD	QD	16	Young	44825	19	2.133
12024.2A6QX	QX	16	Young	35217	39	2.528
12024.2A7ND1002	ND	56	Young	44243	43	2.514
12024.2A8NX1002	NX	56	Young	42132	35	1.158
12024.2A9SD1002	SD	61	Young	80265	33	2.457
12024.2B10NX1010	NX	64	Young	34168	58	1.625
12024.2B11SD1002	SD	61	Young	34844	50	2.550
12024.2B1YD2	YD	75	Old	20130	50	2.582
12024.2B3KD1012	KD	112	Old	50199	81	2.727
12024.2B4KX1012	KX	112	Old	37879	82	1.858
12024.2B5MZ1012	MZ	99	Old	46255	58	2.546
12024.2B6YD1009	YD	75	Old	49234	69	2.333
12024.2B7YX1009	YX	75	Old	45866	68	2.049
12024.2B8QX1012	QX	26	Young	39043	23	2.144
12024.2B9ND1010	ND	64	Young	63386	41	2.600
12024.2C10NX1021	NX	75	Old	33721	47	2.309
12024.2C11SD1028	SD	87	Old	34369	68	2.135
12024.2C12JX1013	JX	72	Young	15867	59	2.232
12024.2C1JD1012	JD	71	Young	14153	48	1.701

12024.2C2JX1010	JX	69	Young	51490	54	1.152
12024.2C3KX1027	KX	127	Old	53270	78	2.803
12024.2C4MZ1024	MZ	111	Old	43280	81	2.665
12024.2C5YD1010	YD	76	Old	39441	62	1.984
12024.2C6YX1212	YX	139	Old	33983	50	1.108
12024.2C7QD1022	QD	36	Young	39922	63	1.549
12024.2C8QX1022	QX	36	Young	36972	52	2.325
12024.2C9ND1028	ND	82	Old	60459	78	2.332
12024.2D10SD1108	SD	98	Old	36953	61	1.634
12024.2D11SX1106	SX	96	Old	23078	58	2.373
12024.2D12JD1112	JD	102	Old	12489	68	1.294
12024.2D1JX1026	JX	85	Old	14907	53	1.089
12024.2D2QD1012	QD	26	Young	41531	31	1.714
12024.2D3KD1102	KD	133	Old	49878	68	2.123
12024.2D4KX11021	KX	133	Old	39596	80	2.929
12024.2D5YD1102	YD	99	Old	36845	74	1.590
12024.2D6QD1104	QD	49	Young	52372	65	2.084
12024.2D7QX1103	QX	48	Young	61727	74	1.624
12024.2D8ND1105	ND	90	Old	48847	73	2.540
12024.2D9NX1105	NX	90	Old	54837	83	2.928
12024.2E10QX1106	QX	51	Young	39833	54	1.921
12024.2E11ND1108	ND	93	Old	41802	54	2.035
12024.2E12NX1108	NX	93	Old	18345	81	2.949
12024.2E1JX11081	JX	98	Old	9747	74	1.987
12024.2E2SX1115	SX	105	Old	25165	55	2.093
12024.2E3JD1116	JD	106	Old	29967	47	1.151
12024.2E4JX1113	JX	103	Old	29499	64	1.229
12024.2E5KD1127	KD	158	Old	18321	65	2.408
12024.2E6KX1104	KX	135	Old	15909	67	3.255
12024.2E7YD1106	YD	103	Old	38590	79	1.674
12024.2E8YX1119	YX	116	Old	41629	83	2.493
12024.2E9QD1106	QD	51	Young	67197	56	2.371
12024.2F10SD1119	SD	101	Old	25676	71	2.128
12024.2F11SX1125	SX	115	Old	29767	50	1.645
12024.2F12JD1123	JD	113	Old	11419	74	2.342
12024.2F1SD1110	SD	100	Old	19746	85	3.071
12024.2F2KX1112	KX	143	Old	48875	81	1.673
12024.2F3MZ1110	MZ	128	Old	55933	72	1.384
12024.2F4YD1126	YD	123	Old	36458	63	1.374
12024.2F5YX1124	YX	121	Old	34067	70	2.265
12024.2F6QD1119	QD	64	Young	11582	53	2.829
12024.2F7QX1119	QX	64	Young	44218	53	1.641
12024.2F8ND1119	ND	104	Old	27347	79	2.271

12024.2F9NX1111	NX	96	Old	57709	79	2.563
12024.2G10NX1119	NX	104	Old	17686	60	2.505
12024.2G11SD1119	SD	109	Old	18089	61	1.671
12024.2G12JD1125	JD	115	Old	7081	59	2.762
12024.2G1JX11082	JX	98	Old	16275	74	1.815
12024.2G3MZ1106	MZ	124	Old	42928	61	1.249
12024.2G4MZ1127	MZ	145	Old	27942	73	1.914
12024.2G5YD11022	YD	99	Old	34004	66	1.611
12024.2G6YX1126	YX	123	Old	20905	79	2.541
12024.2G7QD1112	QD	57	Young	20346	48	2.040
12024.2G8QX1113	QX	58	Young	16667	49	2.100
12024.2G9ND1112	ND	97	Old	26923	60	1.690
12024.2H1JX1125	JX	115	Old	9058	82	2.808
12024.2H2YX1113	YX	110	Old	21885	59	2.045

Table S2: The information of culture media in this study.

ID	component	Sterilization method
APT	Yeast extract (7.5g/L),Casein peptone (12.5g/L), Glucose (10g/L),Sodium citrate (5g/L),Thiamine hydrochloride (0.001g/L),Sodium chloride (5g/L),Dipotassium hydrogen phosphate (5g/L),Manganese chloride (0.14g/L), Magnesium sulfate (0.8g/L),Ferric sulfate (0.04g/L),Tween 80 (0.2g/L),liver infusion (2g/L)	Autoclaving
BBL	Peptone (15g/L), Yeast extract (2g/L), Glucose (20g/L), Soluble starch (0.5g/L), Sodium chloride (5g/L), L-cysteine (0.5g/L), Tomato extract (2g/L)	Autoclaving
BCG	Skimmed milk powder (100g/L), Yeast extract (10g/L), Bromocresol green (0.016g/L)	Autoclaving
BS	Peptone (10g/L), liver infusion (5g/L) Beef extract powde r(3g/L), Yeast extract (5g/L), Tryptone of pancreatic casein (8g/L), Soluble starch (0.5g/L), Sodium chloride (1g/L), Dipotassium hydrogen phosphate (1g/L), Potassium dihydrogen phosphate (1g/L), Glucose (10g/L), $FeSO_4.7H_2O$ (0.01g/L), $MnSO_4$ (0.005g/L), L-cysteine (0.5g/L)	Autoclaving
DZ	Tryptone (17g/L), Beef extract powder (3g/L), Yeast extract (5g/L),Bile powder (10g/L), sodium chloride (5g/L) , sodium citrate (1g/L) ammonium ferric citrate (0.5g/L), Seven leaf glycosides (1g/L),Sodium azide (0.25g/L)	Autoclaving
EI	Tryptone(20g/L),Sucrose(5g/L),Gelatin(2.5g/L),Sodium acetate (1.5g/L), Yeast extract (5g/L), Ascorbic acid (0.5g/L), Glucose (5g/L), Lactose (5g/L), Sodium chloride (4g/L)	Autoclaving
GYP	Glucose (10g/L), Peptone (5g/L),Yeast extract (10g/L),Anhydrous sodiumacetate (2g/L),$MnSO_4.7H_2O$ (0.02g/L),$MnSO_4.4H_2O$ (0.001g/L),$FeSO_4.7H_2O$ (0.001g/L),Sodium chloride (0.01g/L)	Autoclaving
HC	Peptone (10g/L), Beef extract powder (8g/L), Yeast extract (4g/L), Glucose(20g/L),Dipotassiumhydrogenphosphate(2g/L) ,Diammonium hydrogen citrate (2g/L) ,Sodium acetate (5g/L), Magnesium sulfate (0.2g/L), Manganese sulfate (0.04g/L), Tween80 (1g/L), Cauliflower (8.5%)	Autoclaving and filtration
HD	Peptone (10g/L), Beef extract powder (8g/L), Yeast extract (4g/L), Glucose (20g/L),Dipotassium hydrogenp hosphate (2g/L) ,Diammonium hydrogen citrate (2g/L) ,Sodium acetate (5g/L), Magnesium sulfate (0.2g/L), Manganese sulfate (0.04g/L), Tween80 (1g/L), Soybean (3%)	Autoclaving

HD1	Peptone (10g/L), Beef extract powde r(8g/L), Yeast extract (4g/L), Glucose (20g/L),Dipotassium hydrogenp hosphate (2g/L) ,Diammonium hydrogen citrate (2g/L) ,Sodium acetate (5g/L), Magnesium sulfate (0.2g/L), Manganese sulfate (0.04g/L), Tween80 (1g/L),Black bean (3%)	Autoclaving
HLB	Peptone (10g/L), Beef extract powde r(8g/L), Yeast extract (4g/L), Glucose (20g/L),Dipotassium hydrogen phosphate (2g/L) ,Diammonium hydrogen citrate (2g/L) ,Sodium acetate (5g/L), Magnesium sulfate (0.2g/L), Manganese sulfate (0.04g/L), Tween80 (1g/L),Carrot (8.5%)	Autoclaving and filtration
HS	Peptone (10g/L), Beef extract powder (8g/L), Yeast extract (4g/L), Glucose (20g/L),Dipotassium hydrogen phosphate(2g/L) ,Diammonium hydrogen citrate (2g/L) ,Sodium acetate (5g/L), Magnesium sulfate (0.2g/L), Manganese sulfate (0.04g/L),Tween80 (1g/L), Sweet potato (2%)	Autoclaving
JD	Peptone(10g/L), Beef extract powder(8g/L), Yeast extract(4g/L), Glucose(20g/L),Dipotassiumhydrogenphosphate(2g/L) ,Diammonium hydrogen citrate(2g/L) ,Sodium acetate(5g/L), Magnesium sulfate(0.2g/L), Manganese sulfate(0.04g/L), Tween 80(1g/L) eggs(3%)	Autoclaving
LBS	Yeast extract(5g/L), Tryptone of pancreatic casein(10g/L), Potassium dihydrogen phosphate(6g/L), Ferrous sulfate(0.034g/L), Magnesium sulfate(0.575g/L), Glucose(20g/L), Sodium acetate(25g/L), Ammonium citrate(2g/L), Manganese sulfate(0.12g/L)	Autoclaving
LD	Peptone(10g/L), Beef extract powder(8g/L), Yeast extract(4g/L), Glucose(20g/L),Dipotassiumhydrogenphosphate(2g/L) ,Diammonium hydrogen citrate(2g/L) ,Sodium acetate(5g/L), Magnesium sulfate(0.2g/L), Manganese sulfate(0.04g/L), Tween 80(1g/L) mung bean(3%)	Autoclaving
M17	Soybean peptone(5g/L), peptone(2.5g/L), Casein peptone(2.5g/L), Yeast extract(2.5g/L), Beef extract powder(5g/L), lactose(5g/L), Sodium ascorbate(0.5g/L), β –glycerophosphate(19g/L), Magnesium sulfate(0.25g/L)	Autoclaving
MC	Soybean peptone(5g/L), Beef extract powde(3g/L), Yeast extract(3g/L), Glucose(20g/L), lactose(20g/L), Calcium carbonate(10g/L), Neutral red(0.05g/L)	Autoclaving
MLS	Peptone(10g/L), Beef extract powder(8g/L), Yeast extract(4g/L), Glucose(20g/L),Dipotassiumhydrogenphosphate(2g/L) ,Diammonium hydrogen citrate(2g/L) ,Sodium acetate(5g/L), Magnesium sulfate(0.2g/L), Manganese sulfate(0.04g/L), Tween 80(1g/L), potato(2%)	Autoclaving

MRS	Peptone(10g/L), Beef extract powder(8g/L), Yeast extract(4g/L), Glucose(20g/L), Dipotassium hydrogen phosphate(2g/L) ,Diammonium hydrogen citrate(2g/L) ,Sodium acetate(5g/L), Magnesium sulfate(0.2g/L), Manganese sulfate(0.04g/L), Tween 80(1g/L)	Autoclaving
NG	Peptone(10g/L), Beef extract powder(8g/L), Yeast extract(4g/L), Glucose(20g/L),Dipotassiumhydrogenphosphate(2g/L) ,Diammonium hydrogen citrate(2g/L) ,Sodium acetate(5g/L), Magnesium sulfate(0.2g/L), Manganese sulfate(0.04g/L), Tween 80(1g/L) pumpkin(2%)	Autoclaving
PG	Peptone(10g/L), Beef extract powder(8g/L), Yeast extract(4g/L), Glucose(20g/L),Dipotassiumhydrogenphosphate(2g/L) ,Diammonium hydrogen citrate(2g/L) ,Sodium acetate(5g/L), Magnesium sulfate(0.2g/L), Manganese sulfate(0.04g/L), Tween 80(1g/L) apple(8.5%)	Autoclaving and filtration
RSJ	Peptone number three(7.5g/L), Yeast extract(7.5g/L), Potassium dihydrogen phosphate(2g/L), Glucose(10g/L), Tomato juice(2.5g/L), Polysorbate monoleate(1g/L)	Autoclaving
SL	Peptone(10g/L),Yeast powder(5g/L), Glucose(20g/L), Diammonium citrate(2g/L), Sodium acetate(25g/L), Magnesium sulfate0.58(g/L), Manganese sulfate(0.15g/L), Ferrous sulfate(0.03/L), Potassium dihydrogen phosphate(6g/L), Tween 80(1ml/L)	Autoclaving
SY	Peptone(10g/L), Beef extract powder(8g/L), Yeast extract(4g/L), Glucose(20g/L),Dipotassiumhydrogenphosphate(2g/L) ,Diammonium hydrogen citrate(2g/L) ,Sodium acetate(5g/L), Magnesium sulfate(0.2g/L), Manganese sulfate(0.04g/L), Tween 80(1g/L) yam(2%)	Autoclaving
TJA	Tomato powder(2.5g/L), Beef extractr(10g/L), Yeast powder(5g/L), Dipotassium hydrogen phosphate(2g/L), Glucose(2g/L), Sodium acetate(5g/L), Tween 80(1g/L), lactose(20g/L)	Autoclaving
TPYR	Acid hydrolysis of casein(10g/L), Soybean peptone(5g/L), Yeast extract(2.5g/L), Glucose(15g/L), L-cysteine(0.5g/L), Dipotassium hydrogen phosphate(2g/L), Magnesium chloride(0.5g/L), Calcium chloride(0.15g/L), Zinc chloride(0.000001g/L), Tween 80(1g/L)	Autoclaving
TPYY	Hydrolyzed casein(10g/L), Plant peptone(5g/L), Yeast powde(2g/L)r, Glucose(5g/L), L-cysteine(0.5g/L), Dipotassium hydrogen phosphate(2g/L), Magnesium chloride(0.5g/L), Zinc sulfate,(0.25g/L) Calcium chloride(0.15g/L), Ferric chloride(0.0001g/L), Tween 80(1g/L)	Autoclaving
TYC	Tryptone(15g/L), Yeast extract(5g/L), L-cysteine(0.2g/L), Sodium sulfite(0.1g/L), Sodium chloride(1g/L), Disodium hydrogen phosphate(0.8g/L), Sodium bicarbonate(2g/L), Anhydrous sodium	Autoclaving

	acetate(12g/L), sucrose(50g/L)	
WSW	Proteose peptone(5g/L), Yeast powder(20g/L), Glucose910g/L), Potassium dihydrogen phosphate(2g/L), Tween 80(0.1g/L)	Autoclaving
XG	Peptone(10g/L), Beef extract powder(8g/L), Yeast extract(4g/L), Glucose(20g/L),Dipotassiumhydrogenphosphate(2g/L) ,Diammonium hydrogen citrate(2g/L) ,Sodium acetate(5g/L), Magnesium sulfate(0.2g/L), Manganese sulfate(0.04g/L), Tween 80(1g/L) mushroom(3%)	Autoclaving
XHS	Peptone(10g/L), Beef extract powder(8g/L), Yeast extract(4g/L), Glucose(20g/L),Dipotassiumhydrogenphosphate(2g/L) ,Diammonium hydrogen citrate(2g/L) ,Sodium acetate(5g/L), Magnesium sulfate(0.2g/L), Manganese sulfate(0.04g/L), Tween 80(1g/L) tomato(8.5%)	Autoclaving and filtration
XL	Peptone(10g/L), Beef extract powder(8g/L), Yeast extract(4g/L), Glucose(20g/L),Dipotassiumhydrogenphosphate(2g/L) ,Diammonium hydrogen citrate(2g/L) ,Sodium acetate(5g/L), Magnesium sulfate(0.2g/L), Manganese sulfate(0.04g/L), Tween 80(1g/L) pear(8.5%)	Autoclaving and filtration
YT	Peptone(10g/L), Beef extract powder(8g/L), Yeast extract(4g/L), Glucose(20g/L),Dipotassiumhydrogenphosphate(2g/L) ,Diammonium hydrogen citrate(2g/L) ,Sodium acetate(5g/L), Magnesium sulfate(0.2g/L), Manganese sulfate(0.04g/L), Tween 80(1g/L) dasheen(2%)	Autoclaving

Table S5: the ANI values (> 0.95) and closest species of genomes from JSpecies.

Sample_ID	the_largest_ANI	JSpecies_Taxonomy	New_ID
WC.M17.D3.5061609.NO.18	0.989	Bacteroides_fragilis	Bacteroides-fragilis-panda-18
WC.M17.D3-metabat2-bin.1	0.96	Clostridioides sp002390935	Clostridioides-sp-panda-1
WC.M17.D3-metabat2-bin.29	0.977	Clostridium butyricum	Clostridium-butyricum-panda29
WC.M17.D3.236947.NO.44	0.959	Enterococcus casseliflavus	Enterococcus-casseliflavus-panda-44
WC.M17.D3-metabat2-bin.9	0.98	Enterococcus devriesei	Enterococcus-devriesei-9
WC.M17.D3.140386.NO.49	0.958	Enterococcus faecium	Enterococcus-faecium-panda-49
WC.M17.D3-metabat2-bin.24	0.973	Enterococcus faecium	Enterococcus-faecium-panda-24
WC.M17.D3.128456.NO.39	0.996	Enterococcus gallinarum	Enterococcus-gallinarum-panda-39
WC.M17.D3-metabat2-bin.6	0.975	Enterococcus gallinarum	Enterococcus-gallinarum-panda-6
WC.M17.D3-metabat2-bin.10	0.98	Enterococcus gilvus	Enterococcus-gilvus-panda-10
WC.M17.D3.124191.NO.181	0.99	Enterococcus hirae	Enterococcus-hirae-panda-181
WC.M17.D3.3988452.NO.5	0.978	Enterococcus phoeniculicola	Enterococcus-phoeniculicola-panda-5
WC.M17.D3-metabat2-bin.21	0.978	Enterococcus phoeniculicola	Enterococcus-phoeniculicola-panda-21
WC.M17.D3.202569.NO.195	0.998	Enterococcus saccharolyticus	Enterococcus-saccharolyticus-panda-195
WC.M17.D3.163691.NO.58	0.966	Enterococcus sp._C1	Enterococcus-sp-panda-58
WC.M17.D3.107823.NO.92	0.967	Enterococcus sp._GMD1E	Enterococcus-sp-panda-92
WC.M17.D3-metabat2-bin.4	0.968	Enterococcus sp001297065	Enterococcus-sp-panda-4
WC.M17.D3-maxbin2-bin.002	0.982	Eubacterium limosum	Eubacterium-limosum-panda-2

WC.MC.D5-maxbin2-bin.001	0.989	Lactobacillus fermentum	Lactobacillus-fermentum-panda-1
Lactobacillus-agilis-panda-92-chr	0.958	Lactobacillus agilis	Lactobacillus-agilis-panda-92-chr
WC.MC.D5.793762.NO.46	0.975	Lactobacillus brevis	Lactobacillus-brevis-panda-46
WC.MC.D5-maxbin2-bin.003	0.976	Lactobacillus brevis	Lactobacillus-brevis-panda-3
WC.MC.D5.2071065.NO.3	0.994	Lactobacillus fermentum	Lactobacillus-fermentum-panda-3
Lactobacillus-murinus-panda-123-chr	0.97	Lactobacillus murinus DSM 20452	Lactobacillus-murinus-panda-123-chr
Lactobacillus-plantarum-panda-53-chr	0.973	Lactobacillus plantarum	Lactobacillus-plantarum-panda-53-chr
WC.MC.D5.102149.NO.55	0.995	Lactobacillus plantarum	Lactobacillus-plantarum-panda-55
WC.MC.D5.107526.NO.44	0.993	Lactobacillus plantarum	Lactobacillus-plantarum-panda-44
WC.MC.D5.141101.NO.4	0.985	Lactobacillus plantarum	Lactobacillus-plantarum-panda-4
WC.MC.D5.145218.NO.53	0.996	Lactobacillus plantarum	Lactobacillus-plantarum-panda-53
WC.MC.D5.153422.NO.12	0.988	Lactobacillus plantarum	Lactobacillus-plantarum-panda-12
WC.MC.D5.171248.NO.7	0.992	Lactobacillus plantarum	Lactobacillus-plantarum-panda-7
WC.MC.D5.184366.NO.8	0.98	Lactobacillus plantarum	Lactobacillus-plantarum-panda-8
WC.MC.D5.185708.NO.13	0.986	Lactobacillus plantarum	Lactobacillus-plantarum-panda-13
WC.MC.D5.249841.NO.26	0.992	Lactobacillus plantarum	Lactobacillus-plantarum-panda-26
WC.MC.D5-metabat2-bin.2	0.99	Lactobacillus plantarum	Lactobacillus-plantarum-panda-02
Lactobacillus-reuteri-panda-167-chr	0.966	Lactobacillus reuteri I5007	Lactobacillus-reuteri-panda-167-chr
WC.MC.D5.208660.NO.9	0.993	Lactobacillus plantarum	Lactobacillus-plantarum-panda-9
Lactobacillus-salivarius-panda-103-chr	0.998	Lactobacillus salivarius str. Ren	Lactobacillus-salivarius-panda-103-chr

WC.MC.D5.270176.NO.5	0.988	Pediococcus acidilactici	Pediococcus-acidilactici-panda-5
WC.MC.D5.331209.NO.11	0.98	Pediococcus acidilactici	Pediococcus-acidilactici-panda-11
WC.MC.D5-maxbin2-bin.005	0.971	Pediococcus acidilactici	Pediococcus-acidilactici-panda-005
YZ.HLB.D3.1897198.NO.1	0.974	Pediococcus acidilactici	Pediococcus-acidilactici-1
WC.MC.D5.134057.NO.10	0.988	Pediococcus lolii	Pediococcus-lolii-panda-10
Pediococcus-pentosaceus-panda-54-chr	0.999	Pediococcus pentosaceus	Pediococcus-pentosaceus-panda-54-chr
WC.M17.D3.4171111.NO.21	0.991	Terrisporobacter glycolicus	Terrisporobacter-glycolicus-panda-21
WC.M17.D3-metabat2-bin.25	0.993	Terrisporobacter glycolicus	Terrisporobacter-glycolicus-panda-25
Weissella-confusa-panda-69-chr	0.983	Weissella confusa	Weissella-confusa-panda-69-chr
WC.M17.D3.1481927.NO.15	0.99	Enterococcus gallinarum EG2	Enterococcus-gallinarum-panda-15
WC.M17.D3.1511813.NO.17	0.968	Enterococcus devriesei DSM 22802 [T]	Enterococcus-devriesei-panda-17
WC.M17.D3.1581995.NO.13	0.994	Enterococcus faecalis TX1302	Enterococcus-faecalis-panda-13
WC.M17.D3.1598080.NO.7	0.968	Enterococcus sp. RIT-PI-f	Enterococcus-sp-panda-7
WC.M17.D3.1723833.NO.6	0.973	Enterococcus gilvus ATCC BAA-350 [T]	Enterococcus-gilvus-panda-6
WC.MC.D5.1644962.NO.1	0.99	Lactobacillus brevis KB290	Lactobacillus-brevis-1
WC.M17.D3-metabat2-bin.3	0.987	Enterococcus canintestini DSM 21207	Enterococcus-canintestini-panda-3

Table S6: the ANI values (< 95%) and taxonomy of genomes from JSpecies. Here showed the identification of TYGS and results of CheckM.

Sample_ID	the_largest_ANI(%)[aligned%]	JSpecies_Taxonomy	TYGS_Taxonomy	Completeness	Contamination	New ID
WC.M17.D3.2830991.NO.8	82[70.46]	Bacteria / Firmicutes / Bacilli / Lactobacillales / Enterococcaceae / Enterococcus	New species	95.12	1.25	*Enterococcus-pandaia-panda8*
WC.M17.D3.155136.NO.89	93.13[91.90]	Bacteria / Firmicutes / Bacilli / Lactobacillales / Enterococcaceae / Enterococcus	Enterococcus innesii	43.1	0	Enterococcus-innesii-panda-89
WC.M17.D3_maxbin2_bin.007	73.14[36.26]	Bacteria / Actinobacteria / Actinomycetia / Actinomycetales / Actinomycetaceae / Trueperella	New species	96.34	2.93	Trueperella-pandaia-panda7
WC.M17.D3_maxbin2_bin.001	80.99[38.37]	Bacteria / Actinobacteria / Thermoleophilia / Solirubrobacterales / Conexibacteraceae / Conexibacter	New species	92.81	0.93	Conexibacter-pandaia-panda1
WC.M17.D3.3114720.NO.20	83.31[62.40]	Bacteria / Firmicutes / Clostridia / Eubacteriales / Peptostreptococcaceae / Clostridioides	*Enterococcus devriesei*	84.55	2.45	Enterococcus-devriesei-panda-20
WC.M17.D3.4317422.	93.45 [79.40]	Bacteria / Firmicutes /	*Eubacterium limosum*	90.76	0.23	

NO.11		Clostridia / Eubacteriales / Eubacteriaceae / Eubacterium / Eubacterium callanderi				
